# Increased tissue modulus and hardness in the TallyHO mouse model of early onset type 2 diabetes mellitus

**DOI:** 10.1371/journal.pone.0287825

**Published:** 2023-07-07

**Authors:** Daksh Arora, Erik A. Taylor, Karen B. King, Eve Donnelly

**Affiliations:** 1 Department of Materials Science and Engineering, Cornell University, Ithaca, New York, United States of America; 2 Sibley School of Mechanical and Aerospace Engineering, Cornell University, Ithaca, New York, United States of America; 3 Department of Orthopedics, University of Colorado School of Medicine, Aurora, Colorado, United States of America; 4 Research Institute, Hospital for Special Surgery, New York, New York, United States of America; Rensselaer Polytechnic Institute, UNITED STATES

## Abstract

Individuals with type 2 diabetes mellitus (T2DM) have a higher fracture risk compared to those without T2DM despite having higher bone mineral density (BMD). Thus, T2DM may alter other aspects of resistance to fracture beyond BMD such as bone geometry, microarchitecture, and tissue material properties. We characterized the skeletal phenotype and assessed the effects of hyperglycemia on bone tissue mechanical and compositional properties in the TallyHO mouse model of early-onset T2DM using nanoindentation and Raman spectroscopy. Femurs and tibias were harvested from male TallyHO and C57Bl/6J mice at 26 weeks of age. The minimum moment of inertia assessed by micro-computed tomography was smaller (-26%) and cortical porosity was greater (+490%) in TallyHO femora compared to controls. In three-point bending tests to failure, the femoral ultimate moment and stiffness did not differ but post-yield displacement was lower (-35%) in the TallyHO mice relative to that in C57Bl/6J age-matched controls after adjusting for body mass. The cortical bone in the tibia of TallyHO mice was stiffer and harder, as indicated by greater mean tissue nanoindentation modulus (+22%) and hardness (+22%) compared to controls. Raman spectroscopic mineral:matrix ratio and crystallinity were greater in TallyHO tibiae than in C57Bl/6J tibiae (mineral:matrix +10%, p < 0.05; crystallinity +0.41%, p < 0.10). Our regression model indicated that greater values of crystallinity and collagen maturity were associated with reduced ductility observed in the femora of the TallyHO mice. The maintenance of structural stiffness and strength of TallyHO mouse femora despite reduced geometric resistance to bending could potentially be explained by increased tissue modulus and hardness, as observed at the tibia. Finally, with worsening glycemic control, tissue hardness and crystallinity increased, and bone ductility decreased in TallyHO mice. Our study suggests that these material factors may be sentinels of bone embrittlement in adolescents with T2DM.

## 1. Introduction

Individuals with type 2 diabetes mellitus (T2DM) have a 1.7-fold greater risk for hip fracture compared to those without diabetes despite having normal to higher dual energy X-ray absorptiometry-derived areal bone mineral density (aBMD) [1]. The higher fracture risk persists in this patient population even after accounting for number of falls, age and body mass index (BMI) [1, 2]. Thus, T2DM might alter other aspects of resistance to bone fracture beyond BMD, such as bone geometry, microarchitecture, and tissue material properties [3]. Additionally, owing to the higher prevalence of T2DM in elderly patients, bone fracture could further worsen mortality and morbidity in these patients.

There is no consensus on the changes in cortical structure and trabecular microarchitecture that occur in patients with T2DM. In subjects with T2DM vs. non-diabetic controls, cortical porosity at the distal radius assessed by high resolution peripheral quantitative tomography (HRpQCT) was reported to be greater in white women (+124%, n = 19/group) [4] and African American women (+26%, n = 22/group) [5] yet in other studies did not differ in postmenopausal white women (n = 30/group) [6], (n = 16/group) [7], and in predominantly white women (n = 129/group) [8]. Cancellous bone at the femoral neck in men with T2DM trended toward greater bone volume fraction (+24%, p = 0.125, 20 T2D, 25 non-T2D) [9] yet did not differ (n = 30/group) [6] and (19 T2D, 13 non-T2D) [10] compared to nondiabetic controls. At the material level, cancellous bone pentosidine was greater (+36%, p < 0.05, 34 T2D, 31 non-T2D) [9] but serum pentosidine did not differ (19 T2D, 13 non-T2D) [10] in men with T2DM compared to non-diabetic controls. However, regression analysis suggested the competing effects of these differences in microarchitecture and material properties in the T2DM group: the trend toward greater BV/TV increased strength, whereas the greater concentration of pentosidine decreased postyield toughness [9]. The effects of T2DM on bone mechanical performance, geometry, microarchitecture, and material properties remains an active area of investigation.

Although the mechanisms that underlie bone fragility in T2DM are not yet well established, hyperglycemia and non-enzymatic collagen crosslinking in the bone matrix are implicated [11]. Glucose impairs osteoblast activities by creating a low pH medium for mineralization, whereas it serves as an energy source that promotes osteoclast activity [12]. Bone resorption and formation markers were reduced in patients with T2DM [13, 14]. The resulting low bone turnover state may increase tissue mineral content [15, 16], stiffness, and hardness [15, 17]; facilitate accumulation of microdamage [16]; and increase bone fragility [16]. Oxidative stress and hyperglycemia promote non-enzymatic collagen crosslinking and result in formation of advanced glycation end products (AGEs), which embrittle bone tissue in vitro and in rodent models by decreasing the postyield displacement and toughness [18, 19]. AGEs could further alter bone quality by inhibiting osteoblast attachment, differentiation, and activity [20].

Because studies in humans that address the mechanisms of fragility in T2DM by directly relating mechanical performance to bone quality have been limited to tissue retrieved from surgery for arthroplasty [9, 10] and cannot address whole-bone properties, mouse models of human disease play a crucial role in filling that gap. No current rodent model of T2DM (reviewed in detail by Fajardo et al. [21]) recapitulates all the changes in the material properties of bone observed in studies of humans with T2DM [22]. With respect to mechanical properties, single-gene mutation models of T2DM (Zucker Diabetic Fatty (ZDF) rat, KK/Ay mouse, and Db/db mouse) generally have reduced structural and tissue-level mechanical performance (Table 1). Polygenic models of T2DM have more variable structural properties, showing enhanced or impaired structural and tissue-level performance depending on the model [22].

**Table 1:** Symbolic summary of the effects of T2DM on bone material properties in humans and rodent models. Each arrow represents the result of one study with compositional, material, or structural outcomes indicated as increased (↑), decreased (↓), and unchanged (↔) vs. non-diabetic controls. Material properties reported here were both directly assessed and estimated from whole-bone tests. Abbreviations: XST = mineral crystallinity; C:P = Carbonate:Phosphate; XLR = collagen maturity; Pen = Pentosidine concentration; E = elastic modulus; σ_y_ = yield stress; σ_ult_ = ultimate stress; K = fracture toughness; P_max_ = maximum load [22].

		Mineral composition			Collagen composition			Material properties					Structural properties	
		Mineral content	XST	C:P	XLR	fAGEs	Pen	E	σ_y_	σ_ult_	Toughness	K	P_max_	Stiffness
	
	Human [9, 10, 23–25]	↑↑↑	↔	↔↑	↔	↑↑/↔↔	↑↑↑	↑/↔/↑	↓/↑	↑/↔	↔↔			
Diet induced obesity	Obese C57BL/6 mice [26–29]	↔	↔	↓/↔		↑	↑	↓/↑	↓↓/↔	↓↓		↓↓/↔	↓↓/↔	↓↓↓
Single gene mutation models	ZDF rats [30–32]	↓/↔↔						↓/↔↔↔	↔	↓/↔↔↔	↔		↓↓↓↓/↔↔	↓↓↓↓/↔↔
	KK/Ay mice [33, 34]	↑	↔	↔	↑		↔						↓	
	Ob/ob mice [35]										↓		↓	↓
	Db/db mice [35, 36]							↓			↓		↓/↔	↓
Polygenic models	ZDSD rats [30, 37–39]	↑↑	↑/↔	↔↔			↔	↓/↔	↔	↓↓/↔↔	↓↓↓/↔	↔	↓	↓
	WBN/Kob rats [19, 40]						↑	↓					↓	↓
	UCD T2DM rat [41]						↑		↓	↓	↓			
	TallyHO mice [42–44]	↑	↔	↓	↑	↔↔	↔	↑/↔↔		↑↑/↔	↓	↔	↑↑/↔↔	↑/↔↔

With respect to compositional properties, TallyHO mice, KK/Ay mice, and ZDSD rats reflect changes in mineral composition observed in human studies, including increased mineral content with maintained or modest changes in crystallinity and carbonate:phosphate ratio, while obese mice and WBN/Kob rats reflect alterations in collagen properties, including increased or comparable AGEs and decreased enzymatic crosslinks (Table 1). Bone tissue from most of the rodent models of T2DM is characterized by higher mineral content compared to controls, suggesting that reduced bone turnover may persist as a common feature of T2DM independent of model-specific pathogenesis.

A key model of early-onset, naturally occurring T2DM and obesity T2DM in humans is the TallyHO mouse. The male TallyHO mice develop T2DM and mimic many characteristics of human T2DM, including hyperglycemia, hyperinsulinemia, and moderate obesity by 10 weeks of age [45]. However, these characteristics are less penetrant in female TallyHO mice [45]. Thus, most research studies of T2DM have used only male mice.

Several models including TallyHO reflect the reduced bone formation rates observed in humans. Reduced osteoblastic and increased osteoclastic markers were observed in the bone marrow of TallyHO mice compared to age-matched C57Bl/6J controls [46] consistent with observations of low bone turnover in humans with T2DM [13]. Reduced bone formation rates in the lumbar vertebrae and femur were also found in the Db/db mouse, a single-gene mutation model of T2DM, and in cancellous bone in the distal femur of C57Bl/6J with high fat diet [21]. Furthermore, femora were stronger and less ductile; had thicker and less porous cortices; and had lower bone volume fraction and thinner, less connected trabeculae at the distal femur compared to SWR controls at 17 weeks of age [42, 43]. However, when non-diabetic TallyHO were used as controls, there were no differences in post-yield displacement, but decrements in cortical and trabecular bone structure were observed [44]. Although the morphology and structural properties of bone in TallyHO mice have been characterized, limited studies have evaluated tissue material properties [42, 43]. In one such study, TallyHO mice had greater mineral content, greater collagen maturity, and decreased carbonate:phosphate ratio compared to age-matched SWR controls [42]. The differences in tissue compositional properties suggest that tissue nano-mechanical properties may also differ in TallyHO mice, but to our knowledge, these properties have not been characterized in this mouse model of T2DM.

Therefore, the objective of this study was to perform a comprehensive structural, geometric, microarchitectural, and material characterization of bone in TallyHO mice. We hypothesized that T2DM would increase strength at the whole-bone level, as well as increase hardness and modulus at the tissue level in TallyHO mice compared to age-matched C57Bl/6J controls.

## 2. Materials and methods

### 2.1 Animal strains, care, and tissue collection

TallyHO/Jng (*n* = 10) and C57Bl/6J (*n* = 5), male mice were purchased from Jackson Laboratory (Bar Harbor, ME) at 8 weeks of age, raised in ventilated cages at 20°C to 22°C with a 14-hour light-dark cycle, and given free access to standard irradiated chow (2920x; Harlan Laboratories, Inc., Indianapolis, IN, USA). Female mice were not included in the study because they do not develop T2DM [45]. Due to the polygenic inheritance of type 2 diabetes in TallyHO mice, an ideal genetic control strain for TallyHO mice does not exist [47]. The C57BL/6 strain has been used as non-diabetic controls in other published reports. [46, 48].

After 8 weeks of age, the animals were weighed weekly, and their day time non-fasting glucose levels were measured weekly until euthanasia. The femora used here were the nonoperative controls from a study of the effects of T2DM on osteoarthritis progression. At 17 weeks of age, mice within each group (TallyHO: n = 10, C57Bl/6J: n = 5) were anesthetized and chosen at random to undergo either a destabilization medial meniscus (DMM) or sham procedure on the left distal femur [49]. The right femora and tibias used in the current study were not operated or otherwise treated. A minimum of three blood glucose measurements (HbA1c%) were performed via tail nick test (Glucose Test Strips; Ascensia Diabetes Care Inc, Parsippany, NJ, USA) for all the mice at 26 weeks of age. TallyHO mice that did not maintain a minimum non-fasting glucose >250 mg/dL (n = 2) were excluded from the analysis. All mice were euthanized with carbon dioxide at 26 weeks of age, and the femora and tibias were dissected. The right femora and tibias were harvested, wrapped in PBS-soaked gauze, and stored at –20°C prior to analysis. All animal care and procedures were performed at the University of Colorado School of Medicine with the approval of the Institutional Animal Care and Use Committee.

### 2.2 Microcomputed tomography

The total bone length was measured from the greater trochanter to the lateral condyle with digital calipers. The right femora were imaged by a microcomputed tomography (μCT) scanner (μCT40; Scanco Medical AG, Brüttisellen, Switzerland; 55 kVp, 145 μA, 400 ms integration time) with an isotropic voxel size of 6 μm. In each femur, two volumes of interest (VOIs) were analyzed: 1) a cortical region centered at the midshaft extending 2.5% of total bone length and 2) a cancellous region in the distal metaphysis of the femur proximal to the growth plate extending 10% of the total bone length and manually contoured to exclude the cortical shell. For the cortical analysis, the images were processed using image analysis software (BoneJ, version 1.4.3; http://imagej.net/BoneJ) with a Gaussian filter to remove noise and thresholded to segment mineralized and unmineralized tissue [50]. The following femoral cross-sectional parameters were calculated: total area (Tt.Ar); cortical area (Ct.Ar); cortical thickness (Ct.Th); marrow area (Ma.Ar); minimum and maximum moments of inertia (I_min_, I_max_); cortical tissue mineral density (Ct.TMD); and cortical porosity (Ct.Po). For the trabecular analysis, images were processed using Scanco Image Processing Language Software. Measurable outcomes for cancellous regions included bone volume fraction (BV/TV), trabecular thickness (Tb.Th), trabecular separation (Tb.Sp), connectivity density (Conn.D), structural model index (SMI), and trabecular tissue mineral density (Tb.TMD).

### 2.3 Mechanical testing

Prior to testing, all bones were thawed to room temperature and kept moist in PBS. The right femora were placed in three-point bending fixtures with a span of 7 mm, oriented with the posterior aspect to be loaded in tension, preloaded to a 2 N compressive load, and loaded to failure at a displacement rate of 0.05 mm/s with an electrically actuated uniaxial load frame (LM1 Testbench, TA Instruments, MN, USA). Force and displacement were measured with a 200-N load cell at a 100-Hz sampling rate. The following mechanical properties were calculated: maximum load, bending stiffness, post-yield displacement, and work to fracture [51]. The bending stiffness is related to the bone tissue elastic modulus and mid-diaphyseal geometry by the following equation:

$$K=48\frac{EI_{min}}{L^{3}}$$ (1)

where *K* is the bending stiffness, *E* is the tissue elastic modulus, *I*_*min*_ is the minimum moment of inertia at the mid-diaphysis, and L is the span length [51]. Samples that had irregularities in force versus displacement data associated with motion during testing (3 TallyHO, 0 C57Bl/6J) were excluded from the analysis of post-yield displacement and work to fracture.

### 2.4 Nanoindentation

The right tibias were prepared for nanomechanical testing. They were manually cleaned of soft tissue, dehydrated with graded ethanols, and embedded in polymethylacrylate (PMMA). A 2-mm-thick transverse mid-diaphyseal section from each tibia was polished anhydrously [52]. Non-contact atomic force microscopy was used to characterize the surface topography of the cortical bone from each tibia for nanoindentation. In each section, the local roughness was measured over four randomly selected 5 x 5 μm^2^ areas to achieve a final maximum RMS roughness of 16 nm. Prior to testing, samples were rehydrated for 2 hours in 99% saturated Hank’s Balanced Salt Solution (HBSS) and were weighed at multiple time points until a plateau in the weight values was reached to ensure complete rehydration for each sample. Samples were then removed from the solution for testing and weighed after the testing was complete (change in sample weight immediately before and after testing < 5% for all samples).

The areas of interest for nanoindentation were chosen by looking at prior dynamic histomorphometry at the mid-diaphysis of tibias. In tibias of C57Bl/6J mice, formation of new bone occurred near the endosteal edge in the anterior-lateral and posterior-medial quadrants at 26 weeks of age [53]. Thus, two cortical quadrants in each sample, anterior-lateral and posterior-medial, were chosen as the areas of interest and characterized with nanoindentation (S1 Fig). Within each quadrant, indents were made in three cortex regions during a single session of indentation: endosteal, intracortical, and periosteal (S1 Fig). Bone microstructure (lamellar and non-lamellar) was visually identified from optical micrographs when choosing the area of interest for nanoindentation (S1 Fig). A scanning nanoindenter (TriboIndenter, Hysitron, Eden Prairie, MN) with a Berkovich diamond tip was used to collect force–displacement data. Before testing, the tip shape was characterized using the method proposed by Oliver and Pharr: a series of indentations were made in a fused silica calibration specimen (E = 72 GPa) [54]. For testing, the tip was loaded into the sample at 100 μN/s, held at the maximum load of 1000 μN for 30s, and unloaded at 100 μN/s. In each cortex region, three indents spaced 5 μm apart were made along a line parallel to the periosteum (S1 Fig). The reduced modulus and hardness were calculated from the unloading portion of each force–displacement curve [55].

### 2.5 Raman spectroscopy

Raman spectra were collected adjacent to indentations in endosteal, intracortical, and periosteal regions to spatially match tissue level mechanical performance with chemical composition. Spectra were collected using a confocal Raman microscope (Alpha300R, WiTec) through a 50x, 0.55 NA long-working-distance objective (Zeiss) using a 785nm 74mW laser with circular polarization. At each point, ten accumulations with 6-s integration times were collected and averaged to generate individual point spectra. Raman spectral analysis was performed using a combination of chemical imaging software (Project 5.2, WiTec) and custom code (MATLAB, The Mathworks). In chemical imaging software, spectra were truncated from 280–2000 cm^-1^ then baseline corrected with a rolling-circle spectral filter (RCF) method [56]. In custom code, peak area and intensity ratios were calculated. The mineral:matrix ratio was calculated as the integrated area of the ν_2_ PO_4_ band (410–460 cm^-1^) to the amide III band (1215–1300 cm^-1^) [57, 58] to minimize the effects of polarization on the collected data. The carbonate:phosphate ratio was calculated as the area of the ν_1_ CO_3_ band (1050–1100 cm^-1^) to the ν_1_ PO_4_ band (930–980 cm^-1^) [59–62] The mineral maturity/crystallinity (MMC) was calculated as the inverse full width at half maximum (FWHM) of the ν_1_ PO_4_ band [62, 63]. The Raman collagen maturity, reflecting alterations in the secondary structure of the collagen matrix, was calculated as the peak intensity ratio at 1660 cm^-1^/ 1690 cm^-1^ [61, 64] directly from baselined spectra, based on previously validated intensity ratios established via second derivative spectroscopy and curve fitting [61, 64] Peak fitting in the amide region was not performed in the current study. At each point, ten accumulations with 6-second integration times were collected and averaged to generate individual point spectra (S5 Fig). In addition, Pentosidine concentration (PEN) was calculated as the intensity ratio at 1495 cm^-1^ normalized to the intensity at CH_2_ (1450 cm^-1^) [61]. The analysis of PEN is considered exploratory because it relies on analysis of small peaks with relatively low SNR (SNR~3 for PEN vs. SNR>10 for other metrics) and is still undergoing validation by gold-standard methods such as HPLC [65]

### 2.6 Statistical analysis

Wilcoxon–Mann–Whitney tests with a significance level of 0.05 were used to compare groups for outcomes of the whole-bone tests and micro-CT analyses. Outcomes of the cortical micro-CT analyses and whole-bone tests were adjusted for body mass to account for functional adaption [66] with a linear regression method [51]. The adjusted outcome was calculated for each mouse, and the slope of the linear regression between the outcome and body mass was calculated separately for each strain [51]. The material properties of the tissue were not adjusted for body mass because they are independent of bone size [67]. Although post-yield displacement is not expected to correlate with body mass, strong relationships between these variables were observed in the current study (S1 and S2 Tables), as has been observed previously [51, 68]. Therefore, adjusted and unadjusted data are presented for post-yield displacement. Outcomes of the cortical micro-CT analyses and whole-bone tests unadjusted for body mass are presented in S3 and S4 Tables respectively.

The nanoindentation and Raman outcomes were analyzed with a linear mixed model with 1) fixed effects of genotype (TallyHO and C57Bl/6J), quadrants (anterior-lateral and posterior-medial), cortex region (endosteal, intracortical, periosteal), and bone microstructure (lamellar and non-lamellar); and 2) a random effect of mouse, quadrant, and cortex region to account for the repeated measures (multiple indents/spectra) collected within each mouse. Normality was confirmed through assessment of the residuals of the linear mixed model, which fell within the Q-Q plot, and homoscedasticity was confirmed using the Levene Test. Multiple comparisons were performed for the fixed effects of genotype and cortex region with a Tukey post-hoc test. All values are expressed as mean ± SD. A significance level of p<0.05 was used for all analyses.

Linear regressions of pooled C57Bl/6J and TallyHO data were performed to examine relationships between tissue level mechanical and compositional parameters. Stepwise selection regressions using the Akaike information criterion with the small sample size correction (AICc), alpha = 0.05, and power > 80% were used to determine which tissue material properties and geometric parameters were the most important determinants of whole-bone mechanical performance. The lifetime-average blood glucose for each mouse was calculated as an average of blood glucose for 16 weeks study period. The relationships of lifetime-average blood glucose with all whole bone mechanical, nanoindentation and Raman spectroscopy outcomes were analyzed to elucidate the effects of hyperglycemia on tissue material properties. For all regressions except work to fracture vs. HbA1c and Raman mineral:matrix vs. HbA1c, in which the relationships differed between genotypes (Table 4), TallyHO and C57Bl/6J data were pooled because the relationships did not differ between genotypes (p > 0.05).

## 3. Results

### 3.1 Mouse characteristics

The TallyHO mice were hyperglycemic and had a greater body mass than C57Bl/6J age-matched controls. The serum HbA1c levels of the TallyHO mice were 98% higher compared to controls at the time of euthanasia (26 weeks) (mean ± SD, TallyHO: 9.90 ± 1.70% versus C57Bl/6J: 5.00 ± 0.39%; p < 0.05). Hyperglycemia in TallyHO mice was confirmed over the 16-week study period. The non-fasting glucose of the TallyHO mice was 53% greater than that of controls at 10 weeks of age (TallyHO: 260.12 ± 114.70 mg/dL versus C57Bl/6J: 169.60 ± 10.17 mg/dL; p < 0.05), 108% greater at 17 weeks of age (at DMM surgery): (TallyHO: 379.75 ± 182.20 mg/dL versus C57Bl/6J: 188.20 ± 29.8 mg/dL; p < 0.05), and 187% greater at 26 weeks of age (at euthanasia): (TallyHO: 448.12 ± 166.50 mg/dL versus C57Bl/6J: 155.80 ± 7.95 mg/dL; p < 0.05). TallyHO mice were 16% heavier than controls at the time of euthanasia (TallyHO: 38.80 ± 7.40 g versus C57Bl/6J: 33.80 ± 3.30; p < 0.05). The body mass for the TallyHO mice was either maintained or increased during the duration of the study. When we examined the relationship between HbA1c and body weight in each group (TallyHO & C57BL/6J), we found that HbA1c and body weight negatively correlated when regressions were performed in each group (TallyHO p = 0.002, R^2^ = 0.80, C57Bl/6J p = 0.045, R^2^ = 0.20).

### 3.2 Bone geometry and microarchitecture

**Fig 1:**
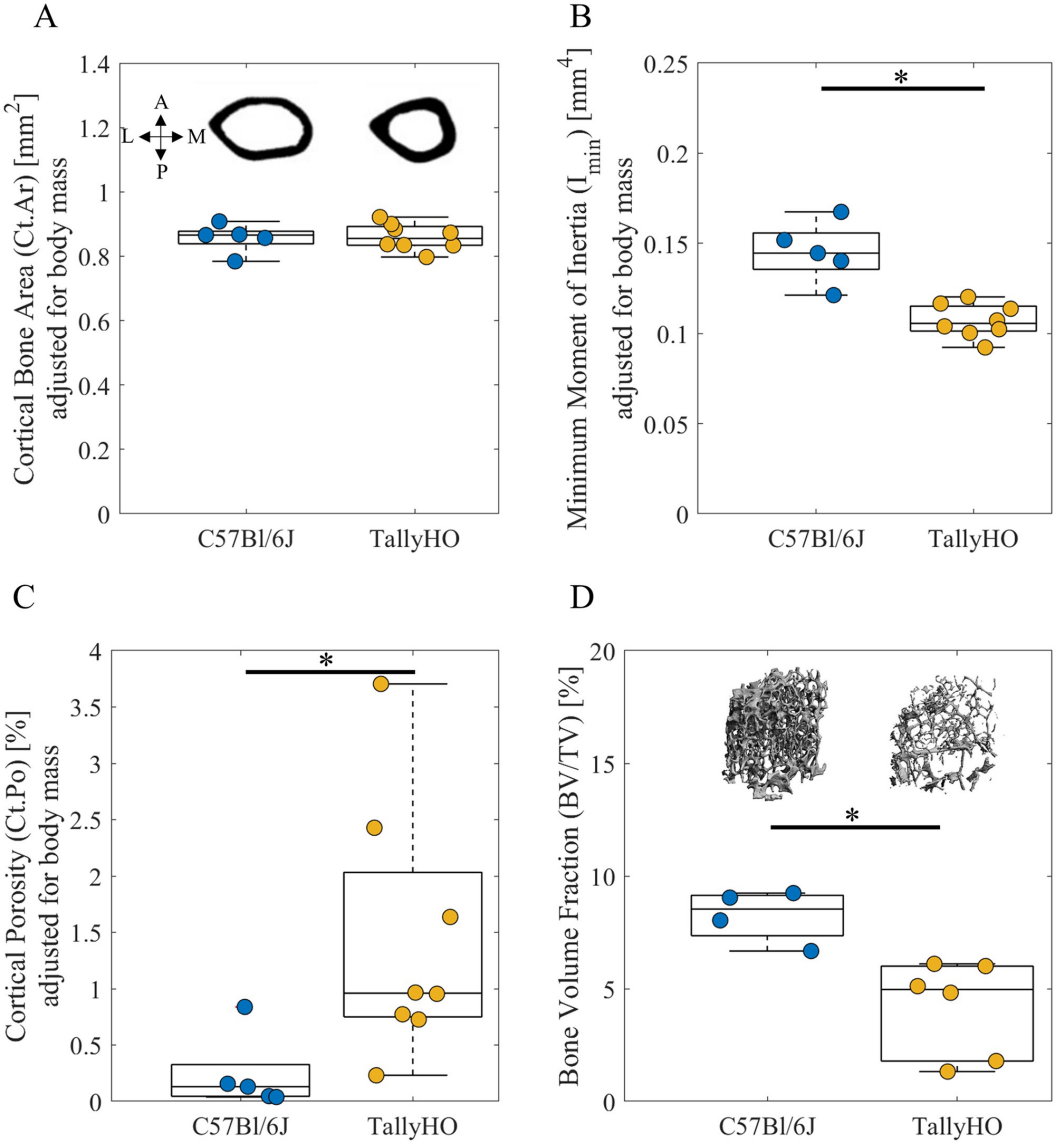
Bone morphology of femur expressed as median ± interquartile range: (A) cortical bone area with representative cross sections at mid-diaphysis showing anatomic directions A: anterior, P: posterior, L: lateral, and M: medial; (B) minimum moment of inertia (I_min_); (C) cortical porosity; (D) bone volume fraction in the distal metaphysis of the femur proximal to the growth plate extending 10% of the total bone length. * p < 0.05 TallyHO vs C57Bl/6J by Wilcoxon–Mann–Whitney test. (A-D: adjusted for body mass).

The minimum moment of inertia was smaller at the mid-diaphysis in femora of TallyHO mice when compared to C57Bl/6J mice after adjusting for body mass (-26%, p < 0.05, Fig 1B) and without adjustment (-22%, p < 0.05, S3 Table). Body mass moderately explained the variance in the minimum moment of inertia (TallyHO, R^2^ = 0.54, p = 0.037; C57Bl/6J, R^2^ = 0.05, p = 0.710, S1 Table). The cortices of TallyHO mice were thicker (+33%, p < 0.05), had similar cortical area, and had greater cortical porosity compared to controls after adjusting for body mass (+490%, p < 0.05) (Table 2, Fig 1A and 1C). Femoral length in TallyHO mice was similar to that of controls (Table 2), and total cross-sectional area (bone + marrow) was smaller (-28%, p < 0.05, Table 2).

**Table 2:** Cortical morphology of the femur at mid diaphysis (adjusted for bone mass), bone volume fraction (BV/TV) (adjusted for body mass), and trabecular microarchitecture of the distal femoral metaphysis expressed as mean ± SD evaluated by micro computed tomography and caliper measurements for femur length. Bold entries indicate p < 0.05 by Wilcoxon–Mann–Whitney test.

Outcome	C57Bl/6J	TallyHO	% difference	p value
	(n = 4–5)	(n = 6–8)	vs C57Bl/6J	
Cortical morphology				
Length (mm)	16.48 ± 0.20	16.29 ± 0.30	-1%	0.476
Tt.Ar (mm^2^)	2.06 ± 0.11	1.48 ± 0.07	-28%	**0.002**
Ma.Ar (mm^2^)	1.20 ± 0.08	0.62 ± 0.06	-48%	**0.002**
Ct.Ar (mm^2^)	0.86 ± 0.04	0.86 ± 0.04	0%	0.943
Ct.Th (mm)	0.19 ± 0.01	0.25 ± 0.01	33%	**0.002**
I_min_ (mm^4^)	0.15 ± 0.02	0.11 ± 0.01	-26%	**0.002**
I_max_ (mm^4^)	0.34 ± 0.04	0.20 ± 0.02	-41%	**0.002**
c (mm)	0.66 ± 0.03	0.59 ± 0.02	-10%	**0.003**
Ct.Po (%)	0.24 ± 0.30	1.43 ± 1.06	490%	**0.011**
Ct.TMD (mg HA/cm^3^)	1159.99 ± 24.29	1227.20 ± 17.97	6%	**0.006**
Trabecular microarchitecture				
BV/TV (%)	9.38 ± 1.06	3.43 ± 1.10	-63%	**0.009**
Tb.Sp (μm)	236.50 ± 19.01	354.50 ± 37.20	50%	**0.010**
Tb.Th (μm)	36.37 ± 1.18	34.93 ± 7.30	-4%	0.748
Tb.N (1/mm)	4.11 ± 0.27	2.85 ± 0.29	-31%	**0.010**
Conn.D (1/mm^3^)	128.10 ± 28.65	50.61 ± 28.85	-60%	**0.019**
SMI	2.34 ± 0.08	2.79 ± 0.33	19%	**0.038**
Tb.TMD (mgHA/cm^3^)	947.19 ± 8.22	969.26 ± 9.70	2%	**0.019**

The volume fraction of trabecular bone in the distal femoral metaphysis was lower in TallyHO mice vs controls (-49%, p < 0.05, Fig 1D). In addition, trabecular separation (+50%, p < 0.05, Table 2) was greater in TallyHO mice compared to controls. No differences in trabecular thickness were observed between the groups (Table 2).

### 3.3 Whole-bone mechanical properties

**Fig 2:**
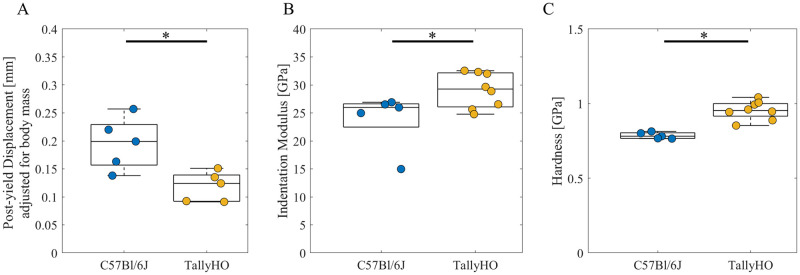
Structural properties of the femur and tissue-level mechanical properties (each data point represents mean across all indents per mouse) expressed as median ± interquartile range: (A) post-yield displacement; (B) indentation modulus; (C) hardness. * p < 0.05 by Wilcoxon–Mann–Whitney test for (A), * *p* < 0.05 Tukey HSD All Pairwise Comparisons for (B) and (C).

The post-yield displacement was 35% lower in the TallyHO mice relative to that in C57Bl/6J controls after adjusting for body mass (p < 0.05, Fig 2A) and had a similar trend without adjustment (-30%, p = 0.15, S4 Table). Body mass explained the variance in the post-yield displacement (TallyHO, R^2^ = 0.82, p = 0.034; C57Bl/6J, R^2^ = 0.59, p = 0.129, S2 Table). Maximum moment, stiffness, and work to fracture were similar between groups after adjusting for body mass (Table 3).

**Table 3:** Structural properties of the femur adjusted for body mass expressed as mean ± SD. Bold entries indicate p < 0.05 by Wilcoxon–Mann–Whitney test.

Whole-bone mechanical properties	C57Bl/6J	TallyHO	% difference	p value
	(n = 5)	(n = 5–8)	vs. C57Bl/6J	
Maximum Moment (N-mm)	32.68 ± 2.12	34.68 ± 2.96	6%	0.430
Stiffness (N/mm)	110.79 ± 11.70	111.63 ± 21.61	1%	0.940
Post-yield displacement (mm)	0.17 ± 0.03	0.11 ± 0.02	-35%	**0.030**
Work to fracture (N-mm)	4.01 ± 0.74	3.38 ± 0.27	-16%	0.300

### 3.4 Tissue-level mechanical properties

The mean tissue indentation modulus and hardness were greater in TallyHO mice compared to that in controls (+22% modulus, +22% hardness, both p < 0.05, Fig 2B and 2C, S5 Table).

**Fig 3:**
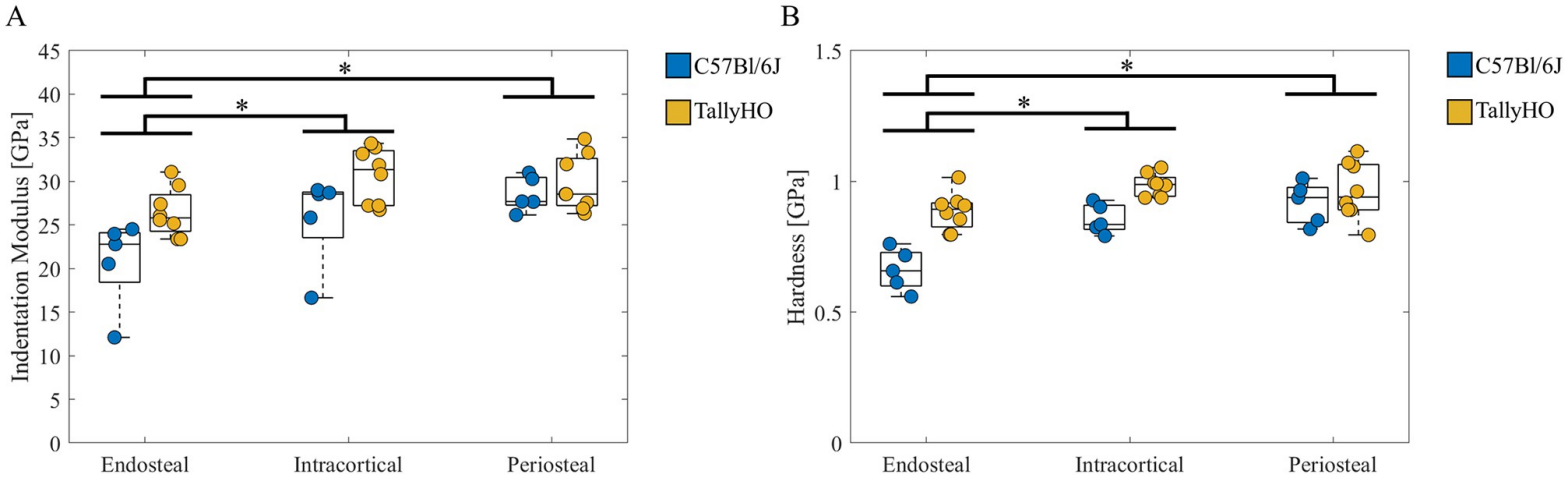
Tissue mechanical properties for each cortex region (each data point represents mean across all indents per mouse) expressed as median ± interquartile range (A) indentation modulus and (B) hardness. * *p* < 0.05 Tukey HSD All Pairwise Comparisons.

No significant interactions between genotype and cortex quadrant were observed for any tissue mechanical properties. Therefore, differences in tissue mechanical properties across quadrants within the cortex regions (endosteal vs intracortical vs periosteal) reported in Fig 3 represent differences across genotypes. The mean tissue indentation modulus was lower in the endosteal bone compared to intracortical or periosteal bone in both the TallyHO and C57Bl/6J mice (-20%: endosteal vs. intracortical; -18%: endosteal vs. periosteal, both p < 0.05, Fig 3A). Similar trends were observed for hardness (-18%: endosteal vs intracortical; -19%: endosteal vs periosteal, both p < 0.05, Fig 3B).

### 3.5 Tissue composition

**Fig 4:**
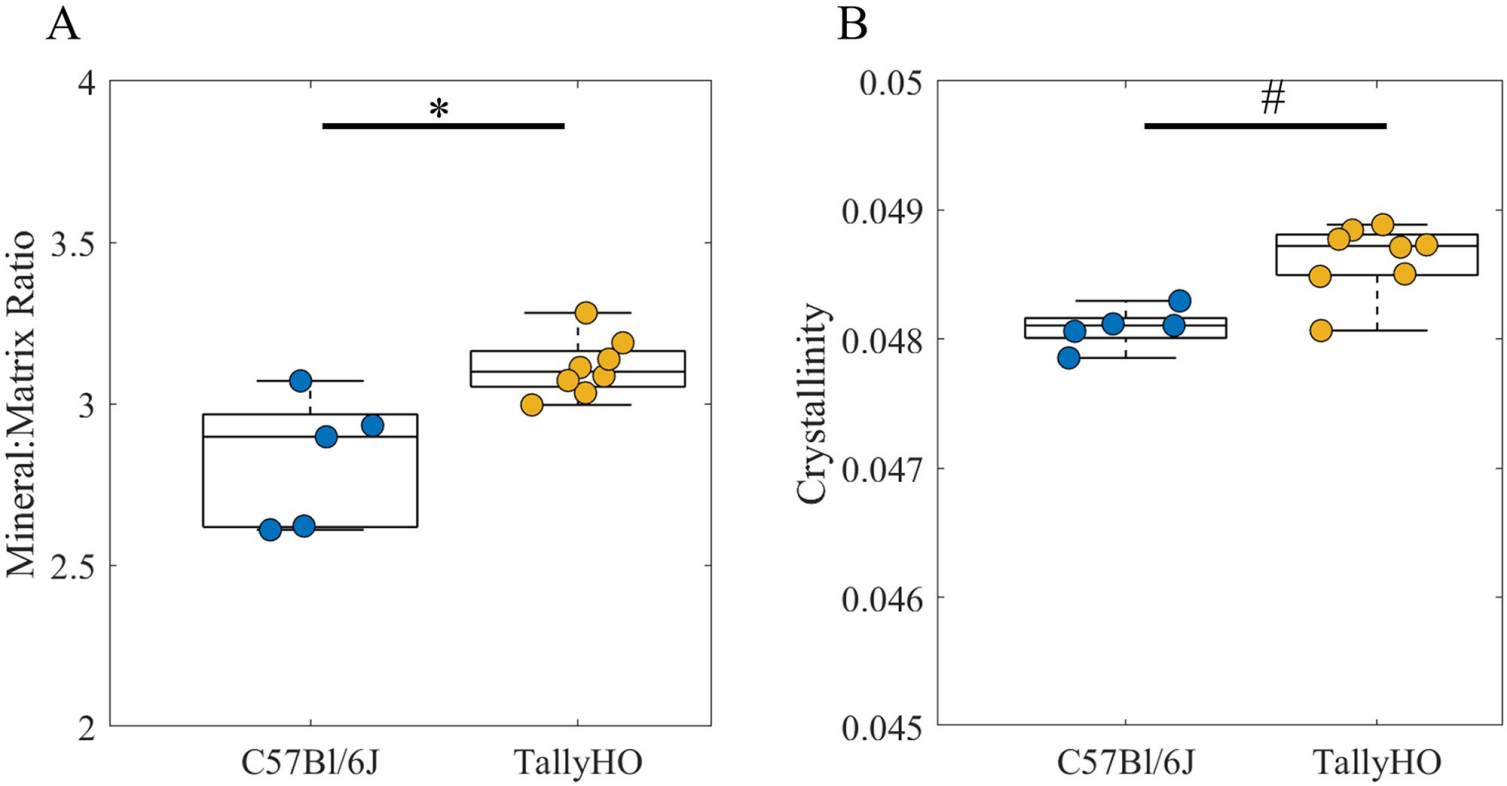
Cortical tissue compositional properties assessed by Raman spectroscopy (each data point represents mean across all spectrum per mouse) expressed as median ± interquartile range: (A) mineral:matrix ratio calculated as the integrated area of the ν_2_PO_4_ band (410–460 cm^-1^) to the amide III band (1215–1300 cm^-1^); (B) Crystallinity calculated as the inverse full width at half maximum (FWHM) of the ν_1_PO_4_ band. * *p* < 0.05 and # p < 0.10 Tukey HSD All Pairwise Comparisons.

The mean Raman mineral:matrix and crystallinity were greater in TallyHO mice compared to that in controls (+10% mineral:matrix, p < 0.05; +0.41% crystallinity, p < 0.1, Fig 4A and 4B, S3 Table). Carbonate:phosphate and collagen maturity did not differ in the femora of TallyHO and C57Bl6/J mice (S5 Table). Pentosidine concentration, which is measure of a specific crosslinking AGE, was numerically greater in TallyHO vs C57Bl6/J mice, but this difference did not reach statistical significance (+19%, p = 0.15) (S5 Table).

Tissue-level mechanical properties correlated with Raman compositional measures. Indentation modulus increased with Raman crystallinity (R^2^ = 0.02 p < 0.05, S2A Fig) and hardness increased with mineral:matrix ratio (R^2^ = 0.04, p < 0.05, S2B Fig).

### 3.6 Relationship of whole-bone mechanical properties to bone geometry and tissue material properties

**Fig 5:**
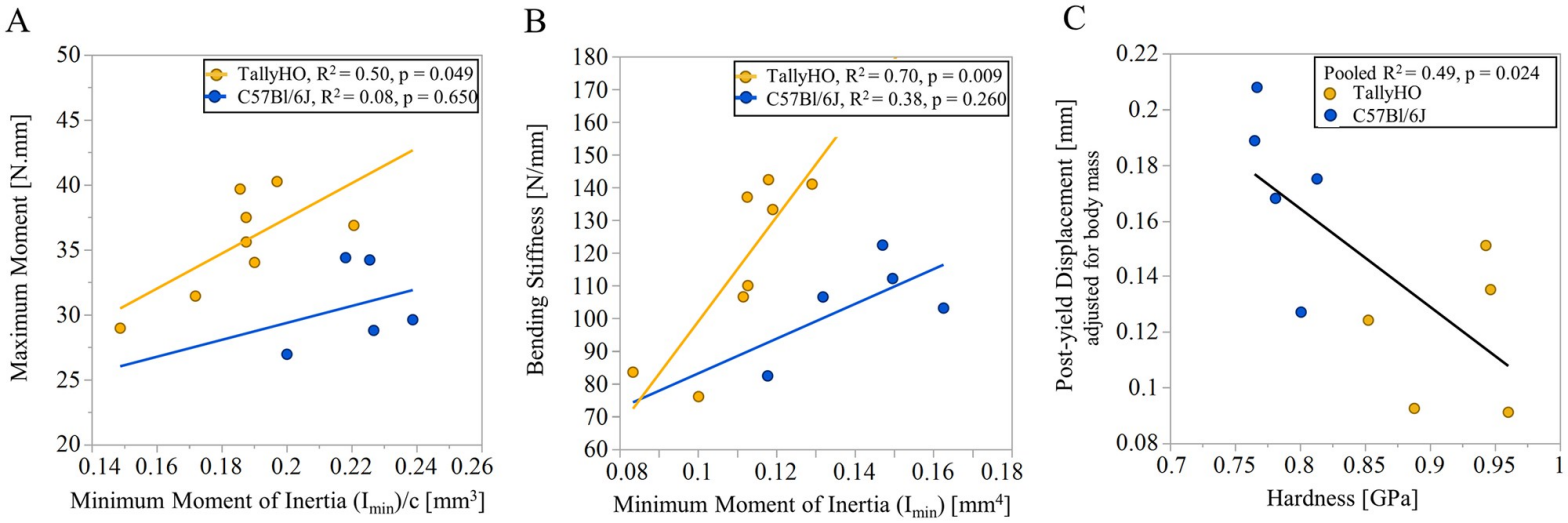
Linear regressions between A) maximum bending moment at failure in a three-point bending test vs. minimum moment of inertia at the mid-diaphysis of the femur (I_min_)/c (ANCOVA slope p = 0.599) B) bending stiffness and minimum moment of inertia (I_min_) at the mid-diaphysis of the femur (ANCOVA slope p = 0.102) C) post-yield displacement and tissue hardness (whole-bone mechanical properties and bone geometry are not adjusted for body mass for regression analysis).

Linear regressions analysis was performed between unadjusted whole-bone mechanical properties, bone geometry, and tissue material properties. As expected, maximum moment increased with section modulus I_min_/c (TallyHO, R^2^ = 0.50, p = 0.049; C57Bl/6J, R^2^ = 0.08, p = 0.650, Fig 5A) and the slopes did not differ between genotypes. Bending stiffness increased with moment of inertia I_min_ (TallyHO, R^2^ = 0.70, p = 0.009; C57Bl/6J, R^2^ = 0.38, p = 0.260, Fig 5B). The regression slope of bending stiffness vs. moment of inertia I_min_ trended to be different between genotypes: TallyHO femora had 201% greater slope vs. controls (p = 0.102, Fig 5B). Post-yield displacement decreased with tissue hardness (R^2^ = 0.49, p < 0.05, Fig 5C). A combination of Raman crystallinity and collagen maturity explained 83% of the observed variation in post-yield displacement (R^2^ = 0.83, p < 0.05, S3 Fig). Specifically, greater values of crystallinity and collagen maturity were associated with lower values of post-yield displacement (R^2^ = 0.83, p < 0.05, S3 Fig). Inclusion of tissue material properties did not improve the regression models for bending stiffness and maximum moment over models with minimum moment of inertia alone, and thus single variable regression models were used to describe the relationship.

### 3.7 Relationship of blood glucose metrics to whole-bone mechanical properties and tissue material properties

Post-yield displacement decreased with increased HbA1c levels (R^2^ = 0.42, p < 0.05; Table 4). Tissue-level hardness increased with lifetime-average blood glucose (R^2^ = 0.37, p < 0.05; Table 4) and HbA1c levels (R^2^ = 0.66, p < 0.05; Table 4). Raman crystallinity increased with lifetime-average blood glucose (R^2^ = 0.34, p < 0.05; Table 4) and HbA1c levels (R^2^ = 0.48, p < 0.05; Table 4).

**Table 4:** Correlations between C57Bl/6J and TallyHO lifetime-average blood glucose and HbA1c with whole-bone mechanical properties adjusted for body mass, nano mechanical properties and Raman compositional properties (Regression slopes for work to fracture vs. HbA1c and Raman mineral:matrix vs. HbA1c differed in C57Bl/6J vs. TallyHO, p < 0.05) correlation coefficients are reported for C57Bl/6J and TallyHO groups combined.

Outcome	Lifetime-average blood glucose level (mg/dL)				HbA1c %		
	Correlation coefficient (r)	*R* ^*2*^	*p*		Correlation coefficient (r)	*R* ^*2*^	*p*
Whole-bone mechanical properties							
Maximum Moment	0.17	0.03	0.567		0.36	0.13	0.214
Stiffness	-0.10	0.01	0.728		0.03	0.00	0.911
Post-yield displacement	-0.62	0.38	0.054		-0.65	0.42	**0.041**
Work to Fracture	-0.48	0.23	0.158	C57Bl/6J	0.78	0.62	0.112
				TallyHO	-0.56	0.31	0.320
Nanomechanical properties							
Indentation Modulus	0.33	0.11	0.269		0.44	0.19	0.134
Hardness	0.61	0.37	**0.028**		0.78	0.66	**0.001**
Raman compositional properties							
Mineral:matrix ratio	0.51	0.26	0.075	C57Bl/6J	0.91	0.82	**0.034**
				TallyHO	-0.01	0.00	0.977
Carbonate:Phosphate	0.04	0.00	0.891		0.08	0.01	0.784
Crystallinity	0.58	0.34	**0.038**		0.70	0.48	**0.008**
Collagen maturity	-0.15	0.02	0.616		-0.21	0.04	0.495

## 4. Discussion

In this study we characterized the structural, geometric, microarchitectural, and tissue material properties of bones from TallyHO mice at 26 weeks of age. Femora in TallyHO mice had smaller total cross-sectional areas and minimum moment of inertia compared to C57Bl/6J controls after adjusting for body mass (Table 2), indicating reduced geometric resistance to bending. However, the cortical bone area did not differ between the groups with and without adjustment for body mass. The difference in cortical geometry suggests that TallyHO mice have reduced periosteal expansion, but similar accumulation of bone mass compared to C57Bl/6J controls. The cortical porosity was greater in the TallyHO femora compared to C57Bl/6J (Table 2). The porosity measurements were however limited by the resolution of the isotropic voxel size of 6 μm, include lacunae (5 μm to 20 μm dimensions), vascular pores (∼10 μm in diameter), and macropores that are visible in μCT images (20 μm to 50 μm in diameter) [51]. At the distal femoral metaphysis in TallyHO mice, several trabecular deficits were evident compared to controls. The trabecular BV/TV was lower, and trabeculae were more separated in TallyHO mice (Table 2). These results are also consistent with prior studies that showed significant loss of trabecular bone in TallyHO mice [42–44]. Excessive bone resorption due to increased osteoclast activities in TallyHO mice not balanced by sufficient bone formation [43] could explain the observed trabecular deficits.

Despite smaller moments of inertia and greater cortical porosity in TallyHO femora, which indicates reduced geometric resistance to bending [67, 69], the strength and stiffness of the TallyHO femora were similar to those of controls after adjusting for body mass (Table 3). Similar trends were observed for data unadjusted for body mass. The whole-bone strength and stiffness were similar in previous studies when compared to SWR/J as controls at 34 weeks of age [42] and when compared to non-diabetic TallyHO as controls at 20 weeks of age [44] after adjusting for body mass. Minimum moment of inertia was the best predictor of whole-bone strength and stiffness but explained 73% of the variability in strength and 68% of the variability in stiffness (Fig 5). The regression slope of bending stiffness vs. minimum moment of inertia, which represents bone tissue elastic modulus (Eq 1), was 201% greater for TallyHO mice than for controls. To understand the mechanisms of altered structural behavior of bone in TallyHO mice, we characterized tissue-level nano-mechanical properties with nanoindentation for the first time in this mouse model, to our knowledge. As hypothesized, TallyHO mice had stiffer and harder cortical bone at the tibial midshaft compared to controls (Fig 2). The maintenance of structural stiffness and strength of TallyHO mouse femora despite reduced geometric resistance to bending could potentially be explained by increased tissue modulus and hardness, as observed at the tibia.

Tibial cortical bone was characterized in two quadrants, anterior-lateral and posterior-medial (S1 Fig). Prior dynamic histomorphometry at the mid-diaphysis of tibiae in C57Bl/6J mice indicates formation of new bone near the endosteal edge in the anterior-lateral and posterior-medial quadrants at 26 weeks of age [53]. The expected effects of PMMA embedding on measured bone material properties in the current study are minimal in the current study. In our study, we rehydrated samples prior to testing to restore hydration critical to anelastic and plastic behavior [54]. We also indented >2 μm from the endosteal edge to avoid including the softer PMMA in the sampling volume [70]. Indentation modulus and hardness did not differ between quadrants (Fig 3). When the effect of cortex region was examined while accounting for bone microstructure type (lamellar or non-lamellar), the endosteal bone was softer and more compliant than intracortical and periosteal bone. These observations are consistent with prior studies that demonstrated that younger bone is softer and more compliant than older bone [71].

Lower reduced modulus determined by nanoindentation was observed at the femoral cortex of Db/db mice vs WT controls, the only other mouse model of T2DM characterized using nanoindentation. However, the Db/db mouse is a single-gene mutation model of T2DM, and altered leptin receptors may play a role in the mechanisms by which tissue material properties are altered [72]. The TallyHO mouse model is polygenic model, and the more pronounced changes in bone material properties in polygenic models likely arise from contributions from multiple genetic mutations, along with systemic effects more representative of T2DM [21]. The first-cycle and total indentation distance from RPI were greater in TallyHO tibiae vs. SWR/J controls, suggesting less tissue-level resistance to indentation [43]. The effect of T2DM on bone material properties of other mouse and rat models are summarized in Table 1 and reviewed in detail by Lekkala et al. [22].

In addition, the femora of TallyHO mice had lower post-yield displacement (-35%) (Fig 2), consistent with the reduced post-yield displacement observed in TallyHO vs. SWR/J controls [42, 43]. However, post-yield displacement was not different when compared to non-diabetic TallyHO controls [44]. The whole-bone post-yield displacement decreased with hardness assessed by nanoindentation (Fig 5), which is a measure of tissue-level resistance to elastic and plastic deformation. The reduced ductility at the whole-bone level could potentially be explained by alterations in the mineral or the matrix properties. In our study, cortical tissue in TallyHO mice had a greater Raman mineral:matrix ratio (Fig 4) (integrated area of the ν_2_ PO_4_ band (410–460 cm^-1^)/amide III band (1215–1300 cm^-1^) (S5 Fig), consistent with greater cortical TMD [43] observed previously, greater cortical crystallinity, and impaired skeletal acquisition in TallyHO mice [46] compared to that in C57BL/6J controls.

The cortical tissue in TallyHO mice has greater mineral:matrix ratio (ν_1_PO_4_/Proline and ν_1_PO_4_/Amide I) when compared to SWR/J controls [42]; however, cortical TMD assessed by μCT did not differ when compared with non-diabetic TallyHO mice [44]. The trend of greater cortical tissue mineralization is also observed in other mouse models of T2DM such as KK/Ay mice (Table 1) although the absolute values differ because the methods used to calculate the mineral:matrix ratio differ across studies. Overall, minimal differences between groups were observed in the Raman metrics of crosslinking of the collagen, whereas greater differences were observed in the mineral properties. Several factors may account for this observation, including the relative insensitivity of Raman outcomes for AGEs, which rely on small peaks, as compared to gold standard metrics like HPLC [65]. Modest differences in collagen crosslinking assessed by FTIR and HPLC are consistent with prior observations in KK/Ay (~14%) [33] and TallyHO (~12%) mice [42].

The higher degree of mineralization observed in TallyHO mice could reduce the post yield properties of bone [73,74]. The regression models in our study showed that combination of tissue mineral crystallinity and collagen maturity explained 83% of the variation in post-yield displacement (S3 Fig). Specifically, greater values of crystallinity and collagen maturity were associated with less post-yield displacement. Crystallinity is a measure of crystal size and perfection. Increased crystal dimensions can induce residual strains to neighboring mineral crystals and collagen molecules [75] and reduce mobility of collagen molecules [76]. Thus, larger crystals are associated with decreased ductility [76] and toughness [77]. Increased collagen maturity (the ratio of mature trivalent to immature divalent enzymatic crosslinks) arising from a reduction in immature cross-links could decrease bone strength and ductility [19, 78]. Finally, accumulation of AGEs could also embrittle bone tissue by decreasing the postyield displacement and toughness [79, 80] although these were not assessed in the current study.

Polygenic models of T2DM such as the TallyHO mouse more accurately reflect the complex mode of inheritance of human T2DM in humans compared to single-gene mutation models. However, a limitation of the polygenic models is that the lack of a littermate control makes strain a possible contributor to the observed differences in bone between non-diabetic and diabetic mice. Due to the polygenic inheritance of type 2 diabetes in TallyHO mice, an ideal genetic control strain for TallyHO mice does not exist [47]. Some studies have used the non-diabetic TallyHO mice as controls (26). This approach has the advantage of maximizing genetic similarity in the controls but has a key disadvantage in study design and power estimation of inherent uncertainty in the numbers of mice that will meet the threshold for inclusion in the control group. Another disadvantage is that a non-diabetic TallyHO mouse is not a healthy control because it still has the genetic predisposition for developing diabetes. Several other controls have previously been used, including SWR/J and C57BL/6. The SWR/J strain has the greatest genetic similarity to the TallyHO strain (86.8% homology) (25,49). The C57BL/6 strain has also been used as non-diabetic controls in other published reports (28,29).

The inverse relationship observed between HbA1c and body weight in each group, observed previously [42], suggests that greater HbA1c may drive a loss in body weight. However, our study was not designed to test this relationship as a causal mechanism. Tissue material properties showed greater correlation (*R*^*2*^) to study-end HbA1c levels than lifetime-average blood glucose levels. The regressions of whole-bone mechanical properties, nanomechanical properties, and Raman compositional properties with lifetime-average blood glucose and HbA1c levels demonstrated that tissue hardness and Raman crystallinity increased whereas ductility decreased with worsening hyperglycemia. The increased tissue hardness with hyperglycemia suggests the tissue mechanical properties are progressively altered with disease status in TallyHO mice. The differences in tissue material properties in TallyHO mice could arise from the direct or indirect effects of the accumulation of AGEs [20, 81]. Thus, the altered tissue material properties observed here, in addition to other factors such as altered bone morphology, greater fall risk, and use of certain anti-diabetic medications [82] may contribute to the complex and multifactorial influences of increased bone fragility observed clinically in patients with T2DM.

Our study has some important limitations and strengths. Due to the opportunistic nature of the study, the modest sample size may have been insufficient to detect additional differences in mechanical properties between the groups. Because the TallyHO strain mice lack littermate controls, genetic differences may contribute to the observed differences in skeletal phenotypes when compared to C57Bl/6J controls. We did not assess histomorphometry or AGEs, which may more completely characterize the mechanism responsible for the changes in the tissue material properties in the tibiae of TallyHO mice beyond the compositional characterization performed in the current study. Finally, material properties were assessed at the tibia to avoid damage from structural testing at the femur; therefore, the extent to which the differences in material properties observed at the tibia are also present in the femur is unknown. Nevertheless, our data contributes in elucidating the mechanisms of altered structural behavior by maintained stiffness and strength and decreased post yield properties in the TallyHO mouse model of T2DM that are not explained by bone geometry. To our knowledge, this work is the first to characterize the nanomechanical properties of bone in the TallyHO mouse model of T2DM.

## 5. Conclusion

The femora from TallyHO mice have a smaller moment of inertia and greater cortical porosity indicating reduced geometric resistance to bending. However, the bending stiffness and maximum moment to failure did not differ between groups after adjusting for body mass suggesting that the femora from TallyHO mice had similar stiffness and strength compared to age-matched controls. In addition, femora from TallyHO mice have a lower post yield displacement suggesting lower ductility compared to age-matched controls. At the tissue level, cortical bone in TallyHO tibia is stiffer and harder compared to age-matched C57Bl/6J controls. The maintenance of structural stiffness and strength of TallyHO mouse femora despite reduced geometric resistance to bending could potentially be explained by increased tissue modulus and hardness, as observed at the tibia. Furthermore, greater Raman mineral:matrix and crystallinity was observed at the tibia in TallyHO mice vs C57Bl/6J mice, consistent with the reduced ductility observed in the femora of the TallyHO mice. The TallyHO mouse mimics many characteristics of human T2DM such as hyperglycemia, moderate obesity and low bone turnover. Our data shows tissue-level hardness and Raman crystallinity increased with worsening hyperglycemia in TallyHO mice and offers insights into material factors that might contribute to bone embrittlement in adolescent T2DM population.

## Supporting information

S1 Fig Representative mid-diaphyseal tibial cross-section showing location of indentations indicated by black triangles (not to scale). Three indentations in each cortical region (endosteal, intracortical and periosteal) parallel to periosteum were made in two cortical quadrants (anterior-lateral (A-L) and posterior-medial (P-M)).
(TIF)

S2 Fig Regression analyses of pooled C57Bl/6J and TallyHO tissue-level data: (A) tissue indentation modulus vs. crystallinity; (B) hardness vs. mineral:matrix ratio. (TIF)

S3 Fig Regression model of post-yield displacement determined from backward stepwise regressions using the Akaike information criterion (AICc) (Post-yield displacement = 0.97–124.73*(crystallinity) -1.3*(Collagen maturity), Model fit R^2^ = 0.83, p = 0.002). (TIF)

S4 Fig Representative μCT image slice of the femoral cortical mid-diaphysis (thresholded). Cortical porosity ~ 6–50 μm in-plane dimension is evident throughout the cortex as black pixels.
(TIF)

S5 Fig Representative Raman spectra of cortical bone showing characteristic mineral and organic matrix peaks. (TIF)

S1 Table Linear regression between cortical morphology of the femur mid diaphysis and body mass. Bold entries indicate p < 0.05.
(DOCX)

S2 Table Linear regression between whole bone mechanical properties of the femur and body mass. Bold entries indicate p < 0.05.
(DOCX)

S3 Table Cortical morphology of the femur mid diaphysis expressed as mean ± SD evaluated by micro computed tomography and caliper measurements of femur length unadjusted for body mass. Bold entries indicate p < 0.05 by Wilcoxon–Mann–Whitney test.
(DOCX)

S4 Table Structural properties of the femur unadjusted for body mass expressed as mean ± SD. Bold entries indicate p < 0.05 by Wilcoxon–Mann–Whitney test.
(DOCX)

S5 Table Nanoindentation and Raman spectroscopy outcomes mean estimates from linear mixed models. Bold entries indicate statistical significance in which the p value was less than 0.05, Tukey HSD All Pairwise Comparisons, linear mixed model.
(DOCX)

S6 Table Supplementary data: Mouse characteristics. (XLSX)

S7 Table Supplementary data: Bone geometry and mechanical properties (Unadjusted for body mass). (XLSX)

S8 Table Supplementary data: Bone geometry and mechanical properties (Adjusted for body mass). (XLSX)

S9 Table Supplementary data: Bone trabecular microarchitectural properties. (XLSX)

S10 Table Supplementary data: Bone tissue material properties. (XLSX)

S11 Table Supplementary data: Bone tissue indentation modulus by cortex region. (XLSX)

S12 Table Supplementary data: Bone tissue hardness by cortex region. (XLSX)

## References

[1] Janghorbani M, Van Dam RM, Willett WC, Hu FB. Systematic review of type 1 and type 2 diabetes mellitus and risk of fracture. Am J Epidemiol. 2007;166: 495–505. doi: 10.1093/aje/kwm106 17575306

[2] Napoli N, Strotmeyer ES, Ensrud KE, Sellmeyer DE, Bauer DC, Hoffman AR, et al. Fracture risk in diabetic elderly men: the MrOS study. 2014; 2057–2065. 10.1007/s00125-014-3289-6 PMC4344350 24908567

[3] Farr JN, Khosla S. Determinants of bone strength and quality in diabetes mellitus in humans. Bone. 2016;82: 28–34. doi: 10.1016/j.bone.2015.07.027 26211989 PMC4679576

[4] Burghardt AJ, Issever AS, Schwartz AV., Davis KA, Masharani U, Majumdar S, et al. High-resolution peripheral quantitative computed tomographic imaging of cortical and trabecular bone microarchitecture in patients with type 2 diabetes mellitus. J Clin Endocrinol Metab. 2010;95: 5045–5055. doi: 10.1210/jc.2010-0226 20719835 PMC2968722

[5] Yu EW, Putman MS, Derrico N, Abrishamanian-Garcia G, Finkelstein JS, Bouxsein ML. Defects in cortical microarchitecture among African-American women with type 2 diabetes. Osteoporos Int. 2014;26: 673–679. doi: 10.1007/s00198-014-2927-7 25398431 PMC4400116

[6] Farr JN, Drake MT, Amin S, Iii LJM, Mccready LK. In Vivo Assessment of Bone Quality in Postmenopausal Women With Type 2 Diabetes. J Bone Miner Metab. 2014;29. doi: 10.1002/jbmr.2106 24123088 PMC3961509

[7] Furst JR, Bandeira LC, Fan WW, Agarwal S, Nishiyama KK, Mcmahon DJ, et al. Advanced glycation endproducts and bone material strength in type 2 diabetes. J Clin Endocrinol Metab. 2016;101: 2502–2510. doi: 10.1210/jc.2016-1437 27115060 PMC4891790

[8] Samelson EJ, Demissie S, Cupples LA, Zhang X, Xu H, Liu C-T, et al. Diabetes and Deficits in Cortical Bone Density, Microarchitecture, and Bone Size: Framingham HR-pQCT Study. J Bone Miner Res. 2018;33: 54–62. doi: 10.1002/jbmr.3240 28929525 PMC5771832

[9] Hunt HB, Torres AM, Palomino PM, Marty E, Saiyed R, Cohn M, et al. Altered Tissue Composition, Microarchitecture, and Mechanical Performance in Cancellous Bone From Men With Type 2 Diabetes Mellitus. J Bone Miner Res. 2019. doi: 10.1002/jbmr.3711 30866111 PMC6650336

[10] Karim L, Moulton J, Van Vliet M, Velie K, Robbins A, Malekipour F, et al. Bone microarchitecture, biomechanical properties, and advanced glycation end-products in the proximal femur of adults with type 2 diabetes. Bone. 2018;114: 32–39. doi: 10.1016/j.bone.2018.05.030 29857063 PMC6141002

[11] Merlotti D, Gennari L, Dotta F, Lauro D, Nuti R. Mechanisms of impaired bone strength in type 1 and 2 diabetes. Nutr Metab Cardiovasc Dis. 2010;20: 683–690. doi: 10.1016/j.numecd.2010.07.008 20934862

[12] McCabe LR. Understanding the pathology and mechanisms of type I diabetic bone loss. J Cell Biochem. 2007;102: 1343–1357. doi: 10.1002/jcb.21573 17975793

[13] Krakauer JC, McKenna MJ, Buderer NF, Sudhaker Rao D, Whitehouse FW, Michael Parfitt A. Bone loss and bone turnover in diabetes. Diabetes. 1995;44: 775–782. doi: 10.2337/diab.44.7.775 7789645

[14] Gerdhem P, Isaksson A, Åkesson K, Obrant KJ. Increased bone density and decreased bone turnover, but no evident alteration of fracture susceptibility in elderly women with diabetes mellitus. Osteoporos Int. 2005;16: 1506–1512. doi: 10.1007/s00198-005-1877-5 15824889

[15] Malluche HH, Porter DS, Monier-Faugere MC, Mawad H, Pienkowski D. Differences in bone quality in low- and high-turnover renal osteodystrophy. J Am Soc Nephrol. 2012;23: 525–532. doi: 10.1681/ASN.2010121253 22193385 PMC3294305

[16] Allen MR, Burr DB. Bisphosphonate effects on bone turnover, microdamage, and mechanical properties: What we think we know and what we know that we don’t know. Bone. 2011. pp. 56–65. doi: 10.1016/j.bone.2010.10.159 20955825

[17] Wang X, Allen MR, Burr DB, Lavernia EJ, Jeremić B, Fyhrie DP. Identification of material parameters based on Mohr-Coulomb failure criterion for bisphosphonate treated canine vertebral cancellous bone. Bone. 2008;43: 775–780. doi: 10.1016/j.bone.2008.05.023 18599390 PMC2622738

[18] Tang SY, Zeenath U, Vashishth D. Effects of non-enzymatic glycation on cancellous bone fragility. Bone. 2007;40: 1144–1151. doi: 10.1016/j.bone.2006.12.056 17257914 PMC4398019

[19] Saito M, Fujii K, Mori Y, Marumo K. Role of collagen enzymatic and glycation induced cross-links as a determinant of bone quality in spontaneously diabetic WBN/Kob rats. Osteoporos Int. 2006;17: 1514–1523. doi: 10.1007/s00198-006-0155-5 16770520

[20] Karim L, Bouxsein ML. Effect of type 2 diabetes-related non-enzymatic glycation on bone biomechanical properties. Bone. 2016;82: 21–27. doi: 10.1016/j.bone.2015.07.028 26211993 PMC4679472

[21] Fajardo RJ, Karim L, Calley VI, Bouxsein ML. A review of rodent models of type 2 diabetic skeletal fragility. J Bone Miner Res. 2014;29: 1025–1040. doi: 10.1002/jbmr.2210 24585709 PMC5315418

[22] Lekkala S, Taylor EA, Hunt HB, Donnelly E. Effects of diabetes on bone material properties. Curr Osteoporos Rep. 2019;17: 455. doi: 10.1007/s11914-019-00538-6 31713179 PMC6986388

[23] Lekkala S, Donnelly E, Sacher SE, Taylor EA, Williams RM, Moseley KF. Increased Advanced Glycation Endproducts, Stiffness, and Hardness in Iliac Crest Bone From Postmenopausal Women With Type 2 Diabetes Mellitus on Insulin. 2023;38: 261–277. doi: 10.1002/jbmr.4757 36478472 PMC9898222

[24] Bucknell A, King KB, Oren TW, Botolin S, Williams A. Arthroplasty in veterans: Analysis of cartilage, bone, serum, and synovial fluid reveals differences and similarities in osteoarthritis with and without comorbid diabetes. J Rehabil Res Dev. 2012;48: 1195. 22234664 10.1682/jrrd.2010.09.0186 PMC4487361

[25] Pritchard JM, Papaioannou A, Tomowich C, Giangregorio LM, Atkinson SA, Beattie KA, et al. Bone mineralization is elevated and less heterogeneous in adults with type 2 diabetes and osteoarthritis compared to controls with osteoarthritis alone. Bone. 2013;54: 76–82. doi: 10.1016/j.bone.2013.01.032 23356988 PMC5096932

[26] Marin C, Papantonakis G, Sels K, van Lenthe GH, Falgayrac G, Vangoitsenhoven R, et al. Unraveling the compromised biomechanical performance of type 2 diabetes- and Roux-en-Y gastric bypass bone by linking mechanical-structural and physico-chemical properties. Sci Rep. 2018;8: 5881. doi: 10.1038/s41598-018-24229-x 29651097 PMC5897570

[27] Ionova-Martin SS, Wade JM, Tang S, Shahnazari M, Ager JW, Lane NE, et al. Changes in cortical bone response to high-fat diet from adolescence to adulthood in mice. Osteoporos Int. 2011;22: 2283–2293. doi: 10.1007/s00198-010-1432-x 20941479 PMC3132390

[28] Kerckhofs G, Durand M, Vangoitsenhoven R, Marin C, Van Der Schueren B, Carmeliet G, et al. Changes in bone macro-and microstructure in diabetic obese mice revealed by high resolution microfocus X-ray computed tomography. Sci Rep. 2016;6: 1–13. doi: 10.1038/srep35517 27759061 PMC5069481

[29] Ionova-Martin SS, Do SH, Barth HD, Szadkowska M, Porter AE, Ager JW, et al. Reduced size-independent mechanical properties of cortical bone in high-fat diet-induced obesity. Bone. 2010;46: 217–225. doi: 10.1016/j.bone.2009.10.015 19853069 PMC4501030

[30] Reinwald S, Peterson RG, Allen MR, Burr DB. Skeletal changes associated with the onset of type 2 diabetes in the ZDF and ZDSD rodent models. Am J Physiol Endocrinol Metab. 2009;296: E765–74. doi: 10.1152/ajpendo.90937.2008 19158319 PMC2670632

[31] Prisby RD, Swift JM, Bloomfield SA, Hogan HA, Delp MD. Altered bone mass, geometry and mechanical properties during the development and progression of type 2 diabetes in the Zucker diabetic fatty rat. J Endocrinol. 2008;199: 379–388. doi: 10.1677/JOE-08-0046 18755885

[32] Hamann C, Goettsch C, Mettelsiefen J, Henkenjohann V, Rauner M, Hempel U, et al. Delayed bone regeneration and low bone mass in a rat model of insulin-resistant type 2 diabetes mellitus is due to impaired osteoblast function. Am J Physiol Metab. 2011;301: E1220–E1228. doi: 10.1152/ajpendo.00378.2011 21900121

[33] Hunt HB, Pearl JC, Diaz DR, King KB, Donnelly E. Bone Tissue Collagen Maturity and Mineral Content Increase With Sustained Hyperglycemia in the KK-Ay Murine Model of Type 2 Diabetes. J Bone Miner Res. 2018;33: 921–929. doi: 10.1002/jbmr.3365 29281127 PMC5935591

[34] Xu F, Dong Y, Huang X, Li M, Qin L, Ren Y, et al. Decreased osteoclastogenesis, osteoblastogenesis and low bone mass in a mouse model of type 2 diabetes. Mol Med Rep. 2014;10: 1935–1941. doi: 10.3892/mmr.2014.2430 25109926

[35] Ealey KN, Fonseca D, Archer MC, Ward WE. Bone abnormalities in adolescent leptin-deficient mice. Regul Pept. 2006;136: 9–13. doi: 10.1016/j.regpep.2006.04.013 16764953

[36] Huang L, You YK, Zhu TY, Zheng LZ, Huang XR, Chen HY, et al. Validity of leptin receptor-deficiency (db/db) type 2 diabetes mellitus mice as a model of secondary osteoporosis. Sci Rep. 2016;6: 1–7. doi: 10.1038/srep27745 27283954 PMC4901274

[37] Hammond MA, Gallant MA, Burr DB, Wallace JM. Nanoscale changes in collagen are reflected in physical and mechanical properties of bone at the microscale in diabetic rats. Bone. 2013;60: 26–32. doi: 10.1016/j.bone.2013.11.015 24269519 PMC3944921

[38] Creecy A, Uppuganti S, Merkel AR, O’Neal D, Makowski AJ, Granke M, et al. Changes in the Fracture Resistance of Bone with the Progression of Type 2 Diabetes in the ZDSD Rat. Calcif Tissue Int. 2016;99: 289–301. doi: 10.1007/s00223-016-0149-z 27209312 PMC4961536

[39] Gallant MA, Brown DM, Organ JM, Allen MR, Burr DB. Reference-point indentation correlates with bone toughness assessed using whole-bone traditional mechanical testing. Bone. 2013;53: 301–305. doi: 10.1016/j.bone.2012.12.015 23274349 PMC3563255

[40] Igarashi C, Maruyama T, Ezawa I, Takei I, Saruta T. WBN/Kob rat: a new model of spontaneous diabetes, osteopenia and systemic hemosiderin deposition. Bone Miner. 1994;27: 133–144. doi: 10.1016/s0169-6009(08)80215-2 7711521

[41] Acevedo C, Sylvia M, Schaible E, Graham JL, Stanhope KL, Metz LN, et al. Contributions of Material Properties and Structure to Increased Bone Fragility for a Given Bone Mass in the UCD-T2DM Rat Model of Type 2 Diabetes. J Bone Miner Res. 2018;33: 1066–1075. doi: 10.1002/jbmr.3393 29342321 PMC6011658

[42] Creecy A, Uppuganti S, Unal M, Clay Bunn R, Voziyan P, Nyman JS. Low bone toughness in the TallyHO model of juvenile type 2 diabetes does not worsen with age. Bone. 2018;110: 204–214. doi: 10.1016/j.bone.2018.02.005 29438824 PMC5878744

[43] Devlin MJ, Van Vliet M, Motyl K, Karim L, Brooks DJ, Louis L, et al. Early-onset type 2 diabetes impairs skeletal acquisition in the male TALLYHO/JngJ mouse. Endocrinology. 2014;155: 3806–3816. doi: 10.1210/en.2014-1041 25051433 PMC4164927

[44] Thrailkill KM, Bunn RC, Uppuganti S, Ray P, Popescu I, Kalaitzoglou E, et al. Canagliflozin, an SGLT2 inhibitor, corrects glycemic dysregulation in TallyHO model of T2D but only partially prevents bone deficits. Bone. 2020;141. doi: 10.1016/j.bone.2020.115625 32890778 PMC7852344

[45] Kim JH, Saxton AM. The TALLYHO Mouse as a Model of Human Type 2 Diabetes. Animal Models in Diabetes Research. Totowa, NJ: Humana Press; 2012. pp. 75–87. doi: 10.1007/978-1-62703-068-7_6 22893402

[46] Won HY, Lee JA, Park ZS, Song JS, Kim HY, Jang SM, et al. Prominent bone loss mediated by RANKL and IL-17 produced by CD4+ T cells in tallyho/JngJ mice. PLoS One. 2011;6: 2–9. doi: 10.1371/journal.pone.0018168 21464945 PMC3064589

[47] Leiter EH. Selecting the “right” mouse model for metabolic syndrome and type 2 diabetes research. Methods Mol Biol. 2009;560: 1–17. doi: 10.1007/978-1-59745-448-3_1 19504239

[48] Kim JH, Stewart TP, Soltani-Bejnood M, Wang L, Fortuna JM, Mostafa OA, et al. Phenotypic characterization of polygenic type 2 diabetes in TALLYHO/JngJ mice. J Endocrinol. 2006;191: 437–446. doi: 10.1677/joe.1.06647 17088413

[49] Glasson SS, Blanchet TJ, Morris EA. The surgical destabilization of the medial meniscus (DMM) model of osteoarthritis in the 129/SvEv mouse. Osteoarthr Cartil. 2007;15: 1061–1069. doi: 10.1016/j.joca.2007.03.006 17470400

[50] Doube M, Klosowski MM, Arganda-Carreras I, Cordelières FP, Dougherty RP, Jackson JS, et al. BoneJ: Free and extensible bone image analysis in ImageJ. Bone. 2010;47: 1076–1079. doi: 10.1016/j.bone.2010.08.023 20817052 PMC3193171

[51] Jepsen KJ, Silva MJ, Vashishth D, Guo XE, Van Der Meulen MCH. Establishing biomechanical mechanisms in mouse models: Practical guidelines for systematically evaluating phenotypic changes in the diaphyses of long bones. J Bone Miner Res. 2015;30: 951–966. doi: 10.1002/jbmr.2539 25917136 PMC4794979

[52] Donnelly E, Baker SP, Boskey AL, Van Der Meulen MCH. Effects of surface roughness and maximum load on the mechanical properties of cancellous bone measured by nanoindentation. Mater Res Soc Symp—Proc. 2004;823: 103–108. doi: 10.1002/jbm.a.30633 16392128 PMC1502375

[53] Pflanz D, Birkhold AI, Albiol L, Thiele T, Julien C, Seliger A, et al. Sost deficiency led to a greater cortical bone formation response to mechanical loading and altered gene expression. Sci Rep. 2017;7. doi: 10.1038/s41598-017-09653-9 28842678 PMC5572735

[54] Lewis G, Nyman JS. The use of nanoindentation for characterizing the properties of mineralized hard tissues: State-of-the art review. J Biomed Mater Res—Part B Appl Biomater. 2008;87: 286–301. doi: 10.1002/jbm.b.31092 18395829

[55] Pharr GM. An improved technique for determining hardness and elastic modulus using load and displacement sensing indentation experiments. J Mater Res. 1992;7: 1564–1583. doi: 10.1557/JMR.1992.1564

[56] Brandt NN, Brovko OO, Chikishev AY, Paraschuk OD. Optimization of the rolling-circle filter for Raman background subtraction. Appl Spectrosc. 2006;60: 288–293. doi: 10.1366/000370206776342553 16608572

[57] Gamsjaeger S, Hofstetter B, Fratzl-Zelman N, Roschger P, Roschger A, Fratzl P, et al. Pediatric reference Raman data for material characteristics of iliac trabecular bone. Bone. 2014;69: 89–97. doi: 10.1016/j.bone.2014.09.012 25245203

[58] Taylor EA, Lloyd AA, Salazar-Lara C, Donnelly E. Raman and Fourier Transform Infrared (FT-IR) Mineral to Matrix Ratios Correlate with Physical Chemical Properties of Model Compounds and Native Bone Tissue. Appl Spectrosc. 2017;71: 2404–2410. doi: 10.1177/0003702817709286 28485618

[59] Gamsjaeger S, Buchinger B, Zwettler E, Recker R, Black D, Gasser JA, et al. Bone material properties in actively bone-forming trabeculae in postmenopausal women with osteoporosis after three years of treatment with once-yearly Zoledronic acid. J Bone Miner Res. 2011;26: 12–18. doi: 10.1002/jbmr.180 20645415

[60] Awonusi A, Morris MD, Tecklenburg MMJ. Carbonate assignment and calibration in the Raman spectrum of apatite. Calcif Tissue Int. 2007;81: 46–52. doi: 10.1007/s00223-007-9034-0 17551767

[61] Taylor EA, Donnelly E. Raman and Fourier transform infrared imaging for characterization of bone material properties. Bone. Elsevier Inc.; 2020. doi: 10.1016/j.bone.2020.115490 32569874

[62] Taylor EA, Mileti CJ, Ganesan S, Kim JH, Donnelly E. Measures of Bone Mineral Carbonate Content and Mineral Maturity/Crystallinity for FT-IR and Raman Spectroscopic Imaging Differentially Relate to Physical–Chemical Properties of Carbonate-Substituted Hydroxyapatite. Calcif Tissue Int. 2021; 1–15. doi: 10.1007/s00223-021-00825-4 33710382

[63] Mandair GS, Morris MD. Contributions of Raman spectroscopy to the understanding of bone strength. Bonekey Rep. 2015;4. doi: 10.1038/bonekey.2014.115 25628882 PMC4296861

[64] Gamsjaeger S, Robins SP, Tatakis DN, Klaushofer K, Paschalis EP. Identification of Pyridinoline Trivalent Collagen Cross-Links by Raman Microspectroscopy. Calcif Tissue Int. 2017;0: 0. doi: 10.1007/s00223-016-0232-5 28246932

[65] Paschalis EP, Verdelis K, Doty SB, Boskey AL, Mendelsohn R, Yamauchi M. Spectroscopic Characterization of Collagen Cross-Links in Bone. J Bone Miner Res. 2001;16: 1821–1828. doi: 10.1359/jbmr.2001.16.10.1821 11585346

[66] Smith LM, Bigelow EMR, Nolan BT, Faillace ME, Nadeau JH, Jepsen KJ. Genetic perturbations that impair functional trait interactions lead to reduced bone strength and increased fragility in mice. Bone. 2014;67: 130–138. doi: 10.1016/j.bone.2014.06.035 25003813 PMC4413452

[67] Cole JH, Van Der Meulen MCH. Whole bone mechanics and bone quality. Clinical Orthopaedics and Related Research. Springer New York LLC; 2011. 469: pp. 2139–2149. 10.1007/s11999-011-1784-3 PMC3126947 21274760

[68] Guss JD, Horsfield MW, Fontenele FF, Sandoval TN, Luna M, Apoorva F, et al. Alterations to the Gut Microbiome Impair Bone Strength and Tissue Material Properties. J Bone Miner Res. 2017;32: 1343–1353. doi: 10.1002/jbmr.3114 28244143 PMC5466506

[69] van der Meulen MC, Jepsen KJ, Mikić B. Understanding bone strength: size isn’t everything. Bone. 2001;29: 101–4. doi: 10.1016/s8756-3282(01)00491-4 11502469

[70] Pathak S, Vachhani SJ, Jepsen KJ, Goldman HM, Kalidindi SR. Assessment of lamellar level properties in mouse bone utilizing a novel spherical nanoindentation data analysis method. J Mech Behav Biomed Mater. 2012;13: 102–117. doi: 10.1016/j.jmbbm.2012.03.018 22842281 PMC4786738

[71] Miller LM, Little W, Schirmer A, Sheik F, Busa B, Judex S. Accretion of bone quantity and quality in the developing mouse skeleton. J Bone Miner Res. 2007;22: 1037–1045. doi: 10.1359/jbmr.070402 17402847

[72] Williams GA, Callon KE, Watson M, Costa JL, Ding Y, Dickinson M, et al. Skeletal phenotype of the leptin receptor-deficient db/db mouse. J Bone Miner Res. 2011;26: 1698–1709. doi: 10.1002/jbmr.367 21328476

[73] Currey JD. Effects of differences in mineralization on the mechanical properties of bone. Philos Trans R Soc Lond B Biol Sci. 1984;304: 509–518. doi: 10.1098/rstb.1984.0042 6142490

[74] Currey JD, Pitchford JW, Baxter PD. Variability of the mechanical properties of bone, and its evolutionary consequences. J R Soc Interface. 2007;4: 127–135. doi: 10.1098/rsif.2006.0166 17254981 PMC2358968

[75] Baig AA, Fox JL, Young RA, Wang Z, Hsu J, Higuchi WI, et al. Relationships among carbonated apatite solubility, crystallite size, and microstrain parameters. Calcif Tissue Int. 1999;64: 437–449. doi: 10.1007/pl00005826 10203421

[76] Yerramshetty JS, Akkus O. The associations between mineral crystallinity and the mechanical properties of human cortical bone. Bone. 2008;42: 476–482. doi: 10.1016/j.bone.2007.12.001 18187375

[77] Wen X-X, Wang F-Q, Xu C, Wu Z-X, Zhang Y, Feng Y-F, et al. Time Related Changes of Mineral and Collagen and Their Roles in Cortical Bone Mechanics of Ovariectomized Rabbits. Vashishth D, editor. PLoS One. 2015;10: e0127973. doi: 10.1371/journal.pone.0127973 26046792 PMC4457815

[78] ME RLM LM M, DB B. The effect of the microscopic and nanoscale structure on bone fragility. Osteoporos Int. 2008;19. doi: 10.1007/S00198-008-0579-1 18317862

[79] Vashishth D, Gibson GJ, Khoury JI, Schaffler MB, Kimura J, Fyhrie DP. Influence of nonenzymatic glycation on biomechanical properties of cortical bone. Bone. 2001;28: 195–201. doi: 10.1016/s8756-3282(00)00434-8 11182378

[80] Garnero P. The contribution of collagen crosslinks to bone strength. Bonekey Rep. 2012;1: 182. doi: 10.1038/bonekey.2012.182 24363926 PMC3868729

[81] Saito M, Marumo K. Effects of Collagen Crosslinking on Bone Material Properties in Health and Disease. Calcif Tissue Int. 2015;97: 242–261. doi: 10.1007/s00223-015-9985-5 25791570

[82] Napoli N, Chandran M, Pierroz DD, Abrahamsen B, Schwartz AV., Ferrari SL. Mechanisms of diabetes mellitus-induced bone fragility. Nat Rev Endocrinol 2016 134. 2016;13: 208–219. doi: 10.1038/nrendo.2016.153 27658727

