# Supplementary figures and images for: Increased tissue modulus and hardness in the TallyHO mouse model of early onset type 2 diabetes mellitus
Source: PLoS One. 2023 Jul 7;18(7):e0287825. doi: 10.1371/journal.pone.0287825 (PMC10328374; doi:10.1371/journal.pone.0287825)

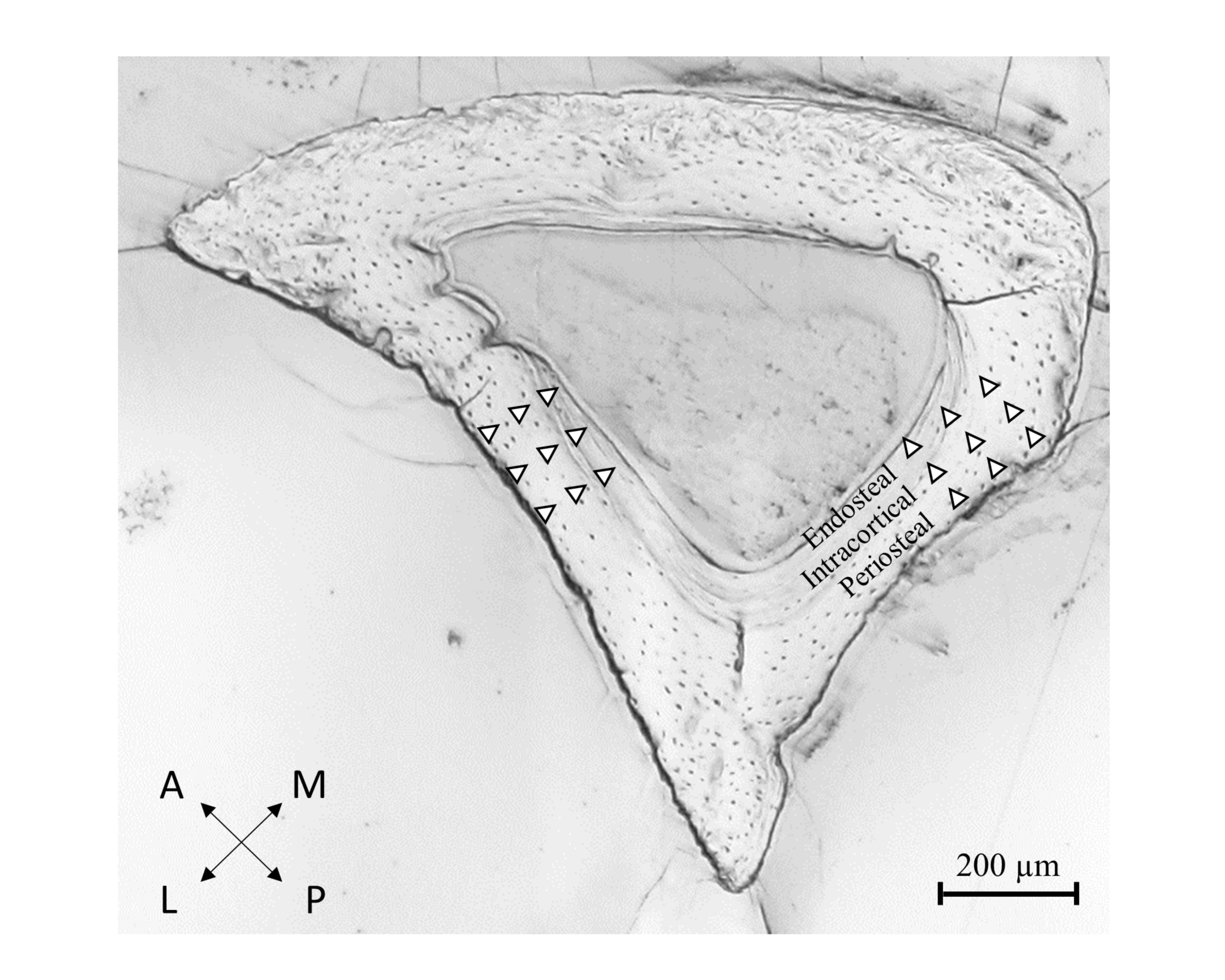

Supplement: S1 Fig — Three indentations in each cortical region (endosteal, intracortical and periosteal) parallel to periosteum were made in two cortical quadrants (anterior-lateral (A-L) and posterior-medial (P-M)). (TIF) [file pone.0287825.s001.tif]

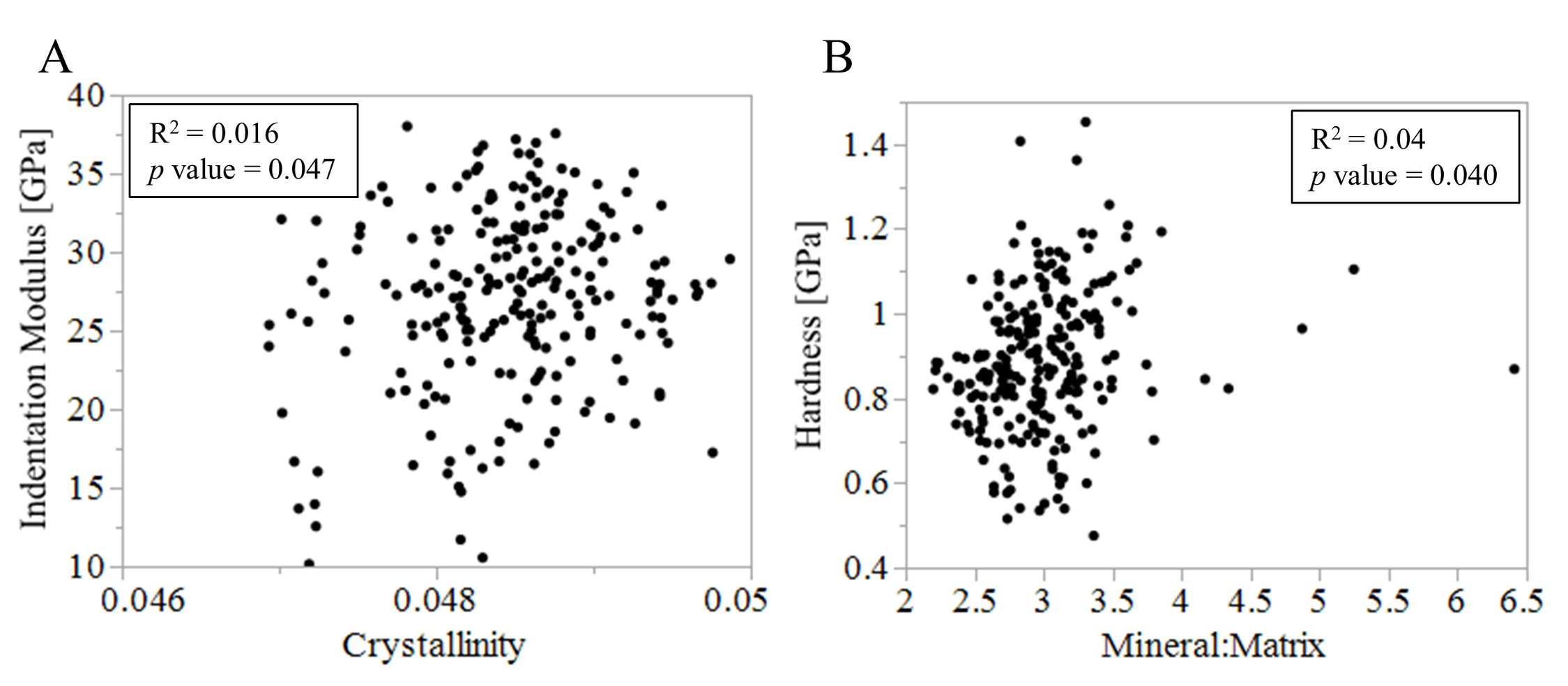

Supplement: S2 Fig — (TIF) [file pone.0287825.s002.tif]

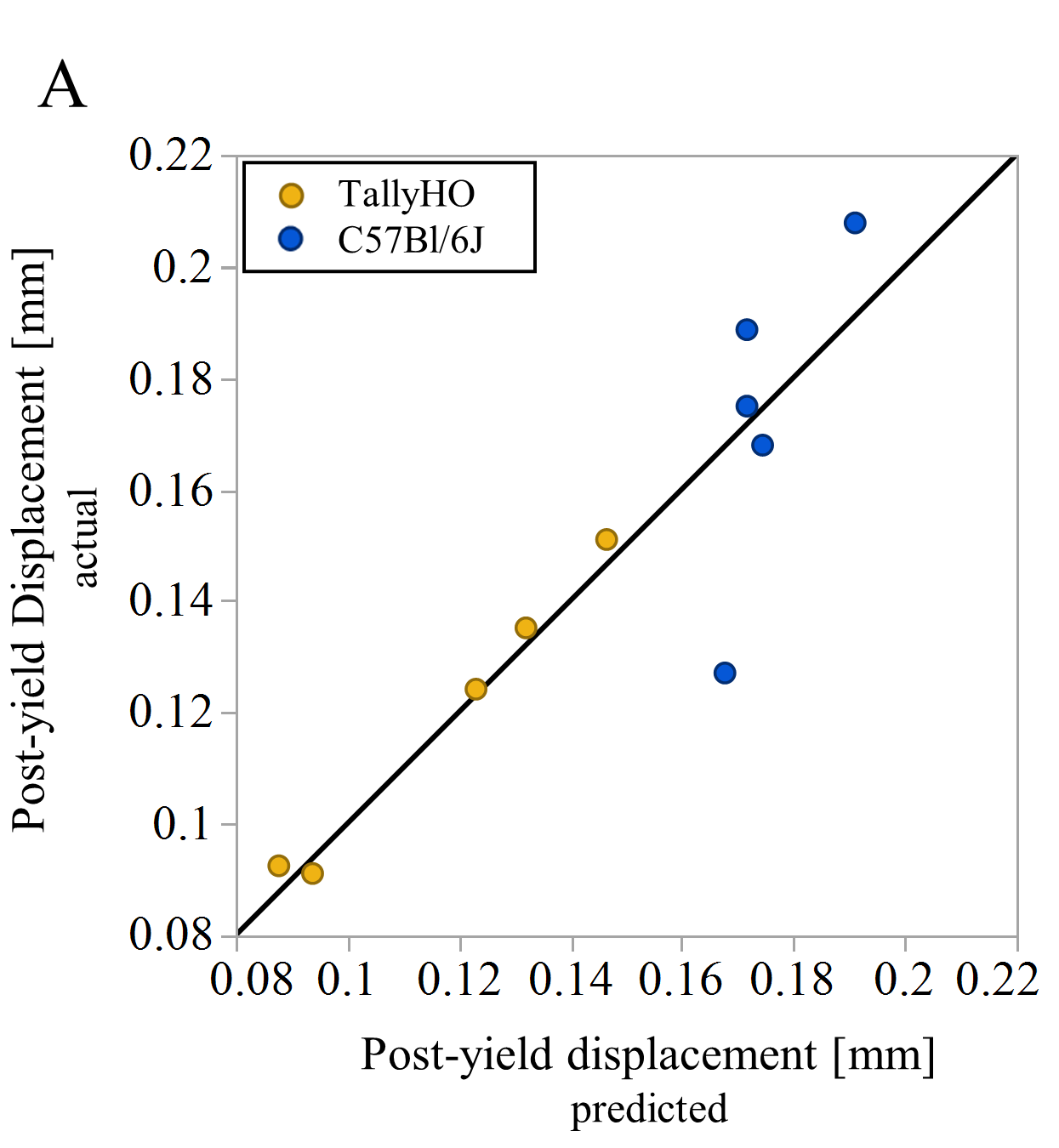

Supplement: S3 Fig — (TIF) [file pone.0287825.s003.tif]

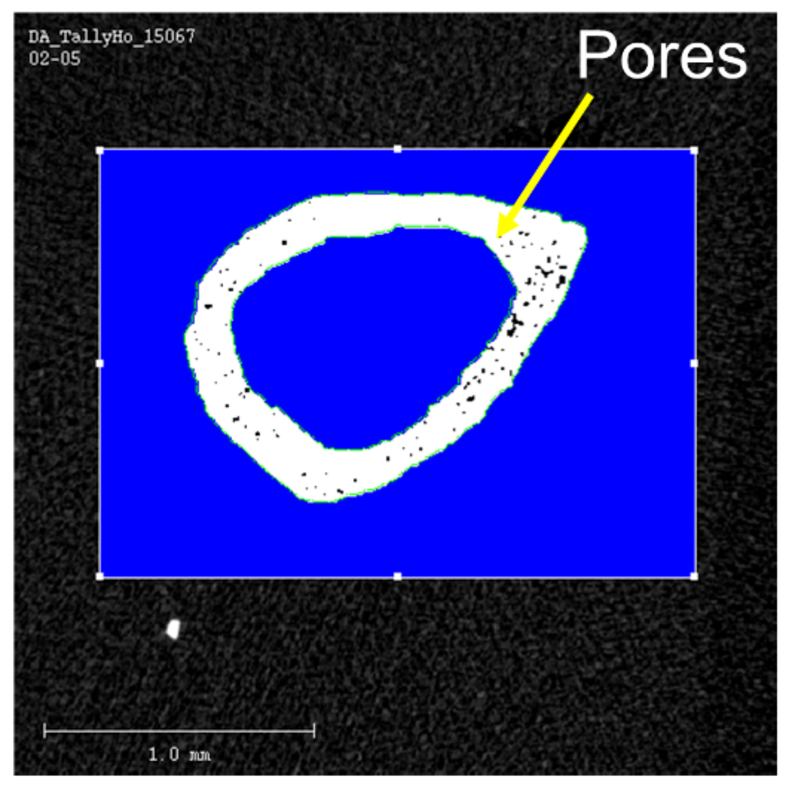

Supplement: S4 Fig — Cortical porosity ~ 6–50 μm in-plane dimension is evident throughout the cortex as black pixels. (TIF) [file pone.0287825.s004.tif]

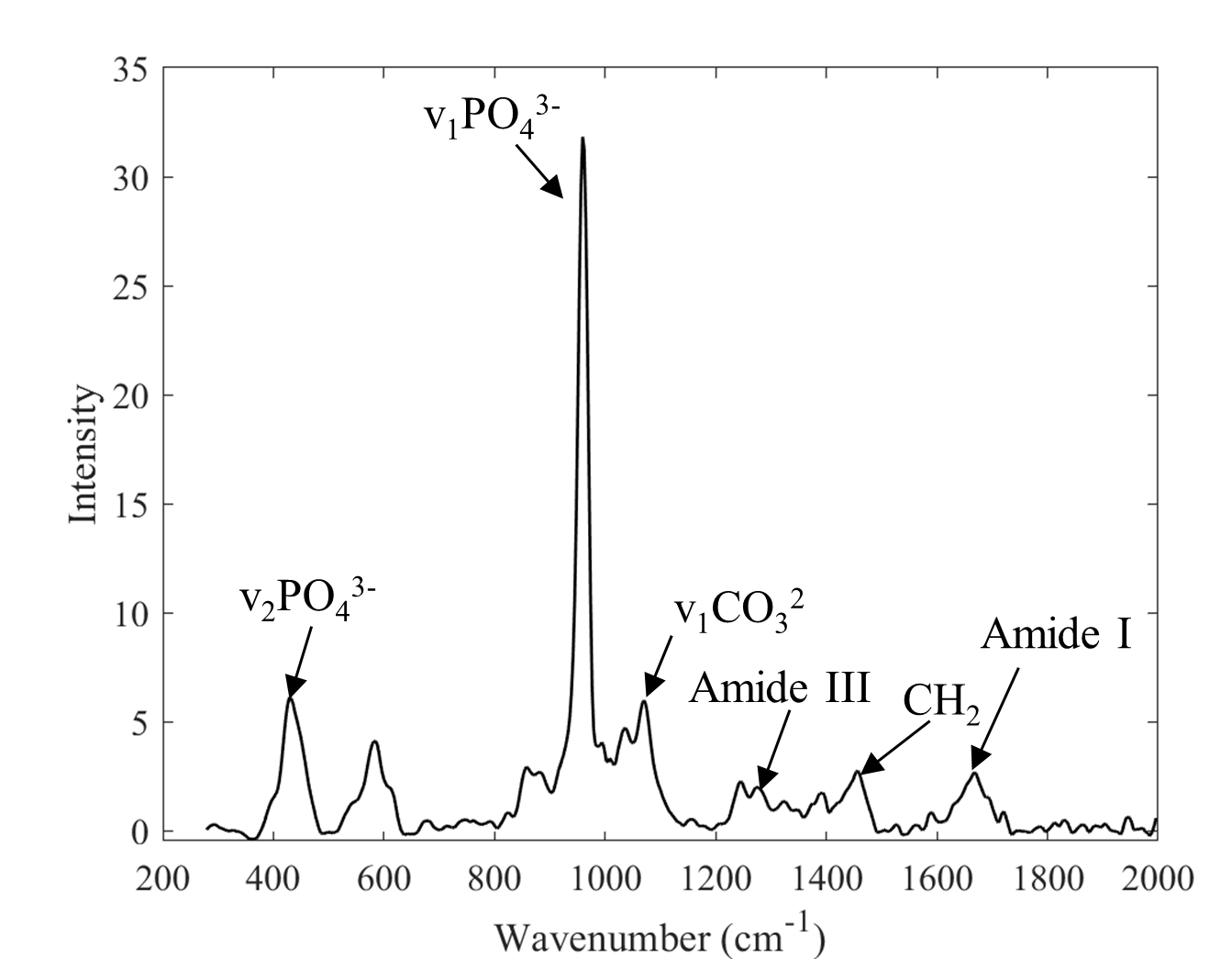

Supplement: S5 Fig — (TIF) [file pone.0287825.s005.tif]
