# Supplementary material for: Increased tissue modulus and hardness in the TallyHO mouse model of early onset type 2 diabetes mellitus
Source: PLoS One. 2023 Jul 7;18(7):e0287825. doi: 10.1371/journal.pone.0287825 (PMC10328374; doi:10.1371/journal.pone.0287825)
Supplement: S1 Table — Bold entries indicate p < 0.05. (DOCX) [file pone.0287825.s006.docx]

**Table S1** Linear regression between cortical morphology of the femur mid diaphysis and body mass. Bold entries indicate p < 0.05

| **Outcome** | **C57Bl/6J**  **(n = 4 - 5)** | | **TallyHO (n = 6 - 8)** | |
| --- | --- | --- | --- | --- |
|  | p value | R^2^ | p value | R^2^ |
| Cortical morphology |  |  |  |  |
| Length (mm) | 0.443 | 0.31 | 0.249 | 0.31 |
| Tt.Ar (mm^2^) | 0.602 | 0.10 | 0.087 | 0.41 |
| Ma.Ar (mm^2^) | 0.504 | 0.16 | 0.931 | 0.00 |
| Ct.Ar (mm^2^) | 0.870 | 0.10 | **0.016** | 0.65 |
| Ct.Th (mm) | 0.623 | 0.09 | 0.126 | 0.35 |
| Imin (mm^4^) | 0.710 | 0.05 | **0.037** | 0.54 |
| Imax (mm^4^) | 0.702 | 0.06 | 0.057 | 0.48 |
| c (mm) | 0.274 | 0.37 | 0.518 | 0.07 |
| Ct.Po (%) | 0.263 | 0.39 | 0.589 | 0.05 |
| Ct.TMD (mg HA/cm^3^) | 0.187 | 0.49 | 0.965 | 0.00 |
| Trabecular microarchitecture |  |  |  |  |
| BV/TV (%) | 0.558 | 0.19 | **0.031** | 0.72 |
