# Supplementary material for: Increased tissue modulus and hardness in the TallyHO mouse model of early onset type 2 diabetes mellitus
Source: PLoS One. 2023 Jul 7;18(7):e0287825. doi: 10.1371/journal.pone.0287825 (PMC10328374; doi:10.1371/journal.pone.0287825)
Supplement: S2 Table — Bold entries indicate p < 0.05. (DOCX) [file pone.0287825.s007.docx]

**Table S2** Linear regression between whole bone mechanical properties of the femur and body mass. Bold entries indicate p < 0.05

| **Whole bone mechanical properties** | **C57Bl/6J**  **(n = 5)** | | **TallyHO (n = 5- 8)** | |
| --- | --- | --- | --- | --- |
|  | p value | R^2^ | p value | R^2^ |
| Maximum Moment (N.mm) | 0.185 | 0.49 | 0.125 | 0.34 |
| Stiffness (N/mm) | 0.434 | 0.21 | 0.232 | 0.22 |
| Post yield displacement (mm) | 0.129 | 0.59 | **0.034** | 0.82 |
| Work to fracture (N.mm) | 0.437 | 0.21 | 0.003 | 0.95 |
