# Supplementary material for: Increased tissue modulus and hardness in the TallyHO mouse model of early onset type 2 diabetes mellitus
Source: PLoS One. 2023 Jul 7;18(7):e0287825. doi: 10.1371/journal.pone.0287825 (PMC10328374; doi:10.1371/journal.pone.0287825)
Supplement: S3 Table — Bold entries indicate p < 0.05 by Wilcoxon–Mann–Whitney test. (DOCX) [file pone.0287825.s008.docx]

**Table S3** Cortical morphology of the femur mid diaphysis expressed as mean ± SD evaluated by micro computed tomography and caliper measurements of femur length unadjusted for body mass. Bold entries indicate p < 0.05 by Wilcoxon–Mann–Whitney test.

| **Outcome** | **C57Bl/6J (n = 4 - 5)** | **TallyHO (n = 6 - 8)** | **% difference vs C57Bl/6J** | **p value** |
| --- | --- | --- | --- | --- |
|  |  |  |  |  |
| Length (mm) | 16.19 ± 0.28 | 16.17 ± 0.39 | 0% | 0.914 |
| Tt.Ar (mm^2^) | 2.02 ± 0.13 | 1.50 ± 0.09 | -26% | **0.002** |
| Ma.Ar (mm^2^) | 1.17 ± 0.09 | 0.62 ± 0.06 | -47% | **0.002** |
| Ct.Ar (mm^2^) | 0.85 ± 0.05 | 0.88 ± 0.04 | 3% | 0.354 |
| Ct.Th (mm) | 0.19 ± 0.01 | 0.26 ± 0.02 | 34% | **0.002** |
| Imin (mm^4^) | 0.14 ± 0.02 | 0.11 ± 0.01 | -22% | **0.010** |
| Imax (mm^4^) | 0.33 ± 0.04 | 0.21 ± 0.02 | -37% | **0.002** |
| c (mm) | 0.64 ± 0.05 | 0.59 ± 0.02 | -7% | 0.220 |
| Ct.Po (%) | 0.26 ± 0.35 | 1.33 ± 1.06 | 408% | **0.033** |
| Ct.TMD (mg HA/cm^3^) | 1177.05 ± 34.66 | 1227.07 ± 19.21 | 6% | **0.030** |
| Trabecular microarchitecture |  |  |  |  |
| BV/TV (%) | 8.25 ± 1.02 | 4.19 ± 1.92 | -49% | **0.010** |
