# Supplementary material for: Increased tissue modulus and hardness in the TallyHO mouse model of early onset type 2 diabetes mellitus
Source: PLoS One. 2023 Jul 7;18(7):e0287825. doi: 10.1371/journal.pone.0287825 (PMC10328374; doi:10.1371/journal.pone.0287825)
Supplement: S4 Table — Bold entries indicate p < 0.05 by Wilcoxon–Mann–Whitney test. (DOCX) [file pone.0287825.s009.docx]

**Table S4** Structural properties of the femur unadjusted for body mass expressed as mean ± SD. Bold entries indicate p < 0.05 by Wilcoxon–Mann–Whitney test

| **Whole bone mechanical properties** | **C57Bl/6J  (n = 5)** | **TallyHO  (n = 5)** | **% difference vs C57Bl/6J** | **p value** |
| --- | --- | --- | --- | --- |
| Maximum Moment (N.mm) | 30.77 ± 2.99 | 35.51 ± 3.66 | 15% | 0.065 |
| Stiffness (N/mm) | 105.26 ± 13.19 | 116.18 ± 24.58 | 10% | 0.354 |
| Post yield displacement (mm) | 0.20 ± 0.04 | 0.14 ± 0.06 | -30% | 0.151 |
| Work to fracture (N.mm) | 4.26 ± 0.82 | 3.81 ± 0.90 | -11% | 0.310 |
