# Supplementary material for: Increased tissue modulus and hardness in the TallyHO mouse model of early onset type 2 diabetes mellitus
Source: PLoS One. 2023 Jul 7;18(7):e0287825. doi: 10.1371/journal.pone.0287825 (PMC10328374; doi:10.1371/journal.pone.0287825)
Supplement: S5 Table — Bold entries indicate statistical significance in which the p value was less than 0.05, Tukey HSD All Pairwise Comparisons, linear mixed model. (DOCX) [file pone.0287825.s010.docx]

**Table S5** Nanoindentation and Raman spectroscopy outcomes mean estimates from linear mixed models. Bold entries indicate statistical signiﬁcance in which the p value was less than 0.05, Tukey HSD All Pairwise Comparisons, linear mixed model

| **Outcome** | **C57Bl/6J (n = 5)** | **TallyHO (n = 8)** | **% difference** | **p value** |
| --- | --- | --- | --- | --- |
|  |  |  | **vs. C57Bl/6J** |  |
| Nanoindentation |  |  |  |  |
| Indentation Modulus | 23.86 | 29.06 | 21.79% | **0.038** |
| Hardness | 0.78 | 0.95 | 21.79% | **<0.0001** |
|  |  |  |  |  |
| Raman Spectroscopy |  |  |  |  |
| Mineral:matrix ratio | 2.82 | 3.10 | 9.93% | **0.005** |
| Carbonate:Phosphate | 0.12 | 0.12 | 1.68% | 0.628 |
| Crystallinity | 0.05 | 0.05 | 0.41% | 0.053 |
| Collagen maturity | 1.39 | 1.37 | -1.44% | 0.417 |
| Pentosidine concentration (PEN) | 0.016 | 0.019 | 18.75 % | 0.146 |
